# A novel H129-based anterograde monosynaptic tracer exhibits features of strong labeling intensity, high tracing efficiency, and reduced retrograde labeling

**DOI:** 10.1186/s13024-021-00508-6

**Published:** 2022-01-10

**Authors:** Hong Yang, Feng Xiong, Hai-Bin Qin, Qun-Tao Yu, Jin-Yan Sun, Hai-Wen Zhao, Dong Li, Youtong Zhou, Fu-Kun Zhang, Xiao-Wen Zhu, Tong Wu, Man Jiang, Xiangmin Xu, Youming Lu, Hong-Jie Shen, Wen-Bo Zeng, Fei Zhao, Min-Hua Luo

**Affiliations:** 1 State Key Laboratory of Virology, CAS Center for Excellence in Brain Science and Intelligence Technology, Center for Biosafety Mega-Science, Wuhan Institute of Virology, Chinese Academy of Sciences, Wuhan, China; 2 University of Chinese Academy of Sciences, Beijing, China; 3 Department of Physiology, School of Basic Medicine and Tongji Medical College, Huazhong University of Science and Technology, Wuhan, China; 4 Chinese Institute for Brain Research, Beijing, China; 5 Changchun Keygen Biological Products Co.Ltd, Changchun, China; 6 Department of Anatomy and Neurobiology, School of Medicine, University of California, Irvine, USA; 7 School of Basic Medical Sciences, Capital Medical University, Beijing, China; 8 Public Health Clinical Center, Fudan University, Shanghai, China

**Keywords:** H129-dgK-G4, Anterograde monosynaptic tracer, Glycoprotein K (gK), Mutant gK pseudotyping, Labeling intensity, Tracing efficiency, Axon terminal invasion, Retrograde labeling, Anterograde specificity, Connectivity quantitation, Alzheimer’s disease

## Abstract

**Background:**

Viral tracers are important tools for mapping brain connectomes. The feature of predominant anterograde transneuronal transmission offers herpes simplex virus-1 (HSV-1) strain H129 (HSV1-H129) as a promising candidate to be developed as anterograde viral tracers. In our earlier studies, we developed H129-derived anterograde polysynaptic tracers and TK deficient (H129-dTK) monosynaptic tracers. However, their broad application is limited by some intrinsic drawbacks of the H129-dTK tracers, such as low labeling intensity due to TK deficiency and potential retrograde labeling caused by axon terminal invasion. The glycoprotein K (gK) of HSV-1 plays important roles in virus entry, egress, and virus-induced cell fusion. Its deficiency severely disables virus egress and spread, while only slightly limits viral genome replication and expression of viral proteins. Therefore, we created a novel H129-derived anterograde monosynaptic tracer (H129-dgK) by targeting gK, which overcomes the limitations of H129-dTK.

**Methods:**

Using our established platform and pipeline for developing viral tracers, we generated a novel tracer by deleting the *gK* gene from the H129-G4. The gK-deleted virus (H129-dgK-G4) was reconstituted and propagated *in* the Vero cell expressing wildtype H129 gK (gK_wt_) or the mutant gK (gK_mut_, A40V, C82S, M223I, L224V, V309M), respectively*.* Then the obtained viral tracers of gK_mut_ pseudotyped and gK_wt_ coated H129-dgK-G4 were tested in vitro and in vivo to characterize their tracing properties.

**Results:**

H129-dgK-G4 expresses high levels of fluorescent proteins, eliminating the requirement of immunostaining for imaging detection. Compared to the TK deficient monosynaptic tracer H129-dTK-G4, H129-dgK-G4 labeled neurons with 1.76-fold stronger fluorescence intensity, and visualized 2.00-fold more postsynaptic neurons in the downstream brain regions. gK_mut_ pseudotyping leads to a 77% decrease in retrograde labeling by reducing axon terminal invasion, and thus dramatically improves the anterograde-specific tracing of H129-dgK-G4. In addition, assisted by the AAV helper trans-complementarily expressing gK_wt_, H129-dgK-G4 allows for mapping monosynaptic connections and quantifying the circuit connectivity difference in the Alzheimer’s disease and control mouse brains.

**Conclusions:**

gK_mut_ pseudotyped H129-dgK-G4, a novel anterograde monosynaptic tracer, overcomes the limitations of H129-dTK tracers, and demonstrates desirable features of strong labeling intensity, high tracing efficiency, and improved anterograde specificity.

**Supplementary Information:**

The online version contains supplementary material available at 10.1186/s13024-021-00508-6.

## Background

Mapping the brain connectome is a key to understand how the brain works, and the development of novel viral tracers with strong labeling intensity, high tracing efficiency, and high specificity is an important task for innovative neurotechnologies. Viral tracers have been broadly applied as a powerful tool to dissect neuronal circuits, which is the basic unit for neural function [1–3]. To analyze the input and output neural networks, both retrograde and anterograde viral tracers are required. The development of anterograde tracers, although are essentially required for mapping output projection, has been lagged behind the development of rabies virus (RABV)-based retrograde tracers [4–7].

Herpes simplex virus 1 (HSV-1) strain H129 (H129), with the feature of predominant anterograde transneuronal transmission, represents a promising candidate for the development of anterograde tracers. HSV-1 is a member of the alpha-herpesvirus subfamily, with a 152 kb DNA genome containing over 70 ORFs [8], which is more complicated and has a larger capacity of genetic payload than RABV (~ 12 kb RNA genome). Thus, there are more difficulties to develop tracer/vector from HSV-1, but the large capacity and the feature of predominant anterograde transneuronal transmission of H129 have attracted scientists to keep working on it.

During the past decade, several polysynaptic anterograde tracers have been developed based on H129 and have already contributed to revealing multiple neuronal circuits in a variety of animal models [7, 9–11]. Besides H129-G4, an anterograde polysynaptic tracer with high labeling intensity for mapping the output network, our group has also developed the first anterograde monosynaptic tracer H129-dTK-tdT [10, 12]. But, its low labeling intensity requires immunostaining for signal amplification to visualize the labeled postsynaptic neurons, which strongly limits its broad application.

To improve the labeling intensity, we added another tdTomato expression cassette to H129-dTK-tdT, thus generated H129-dTK-T2. H129-dTK-T2 can directly visualize the postsynaptic neurons without immunostaining, but the fluorescence labeling intensity remains weak [13]. Both H129-dTK-tdT and H129-dTK-T2 are thymidine kinase (TK) deficient recombinant viruses. TK is essential for HSV-1 genome replication in non-splitting cells, such as neurons [14]. Therefore knockout of the *TK* gene in H129 genome impairs viral genome replication in neurons, and in turn limits the fluorescent protein expression level, which is one of the intrinsic drawbacks of the H129-dTK monosynaptic tracers [12]. An additional drawback of current H129-derived tracers is the potential retrograde labeling caused by axon terminal invasion of virus, resulting in non-anterograde specific labeling and potential misleading of the tracing analysis [12, 15, 16].

To overcome the drawbacks of current H129-derived monosynaptic tracers, we reason that a different strategy is required to develop novel improved ones. The deficiency of the genes associated with viral genome replication, such as *TK*, limits the fluorescent protein expression. Therefore viral gene(s) expressed at the later phase after the viral genome replication, for example, the structural protein genes, is a promising alternative target [12]. Since the structural protein gene deficiency will not influence viral genome replication and viral protein synthesis, the fluorescent protein can be massively expressed, and in turn labels the postsynaptic neuron with strong intensity. To eliminate or reduce the potential retrograde labeling of H129, one possible solution is to modify the viral envelope glycoproteins, inner tegument proteins, or capsid proteins, to impair viral invasion through the axon terminal or the retrograde axonal transportation.

Screening from the viral structural proteins, glycoprotein K (gK) is one of the top candidates. gK, encoded by the *UL53* gene of HSV-1, is a structural component of the virion particle with relatively low abundance [17]. As one of the 12 known envelope glycoproteins, gK plays important roles in virus entry, egress, and virus-induced cell fusion [18–22]. Lack of gK showed mild or no influence on viral genome replication and protein synthesis, but severely impaired the viral transmission among neurons [23]. Differences in the amino acid sequence of gK among different HSV-1 strains also affect the viral entry efficiency, especially the axon terminal invasion and retrograde infection in neurons [23]. Importantly, the relatively low abundance requirement of gK in virions allows the efficient propagation of the gK deficient virus in gK expressing cells, and it also allows the adeno-associated virus (AAV) helper to efficiently compensate gK deficiency without significant influence on the virus assembly/egress/transmission [17]. Taken together, deficiency of gK will not affect viral genome replication and viral protein synthesis, thus resulting in high-level fluorescent protein expression and strong labeling intensity. When gK is compensated by designed AAV helpers, virus propagation and transmission will be efficiently restored, leading to anterograde monosynaptic tracing of the output neurons. Further, mutation of the key amino acids of gK affects viral axon terminal invasion and retrograde infection, so as to limit the retrograde labeling. Thus, target gK meets the demand of developing a novel improved H129-derived monosynaptic anterograde tracer by overcoming multiple drawbacks of the current tracers with designed AAV helpers.

In the present work, the *gK* gene is selected as the knockout target to develop a novel anterograde monosynaptic H129-derived tracer, namely H129-dgK-G4. H129-dgK-G4 can be efficiently reconstituted and propagated in the gK expressing Vero cells, which overcomes the obstacle for H129-dgK-G4 propagation. The AAV helper efficiently compensated gK deficiency for anterograde monosynaptic tracing. Tracing results demonstrated that H129-dgK-G4 monosynaptically transmitted to the postsynaptic neurons and labeled them with bright GFP signals both in vitro and in vivo in a helper-dependent manner. H129-dgK-G4 tracer pseudotyped with a mutant gK (gK_mut_) displayed dramatically reduced retrograde labeling efficiency, making it a tracer with stronger anterograde-specificity. Moreover, H129-dgK-G4 was capable of quantitative analysis for circuit connectivity.

In brief, we have generated a novel anterograde monosynaptic tracer H129-dgK-G4. It has improved tracing property with stronger labeling intensity, higher tracing efficiency, and better anterograde specificity. This novel H129-dgK-G4 represents an improved anterograde monosynaptic tracer for mapping neural projection, and can greatly facilitate brain connectome deciphering, especially in discovering new neural circuits or revealing unknown anatomical connections in known circuits.

## Methods

### Cells and cell culture

Vero-E6 cell (Vero, ATCC#CRL-1586) was obtained from ATCC, maintained in our laboratory, and tested to be mycoplasma-free. The Vero cell lines stably expressing wildtype (gK_wt_) or mutant glycoprotein K (gK_mut_) of H129, namely Vero-gK_wt_ and Vero-gK_mut_, respectively, were generated by lentivirus transduction. Briefly, gK_wt_ coding gene of H129 (Genebank GU734772.1) was cloned from H129-G4 [10] by PCR (gK_wt_ primers: F - TCG AGG AGA ATC CTG GCC CAA TGC TCG CCG TCC GTT CCC TG, R - TCC GAT TTA AAT TCG AAT TCT CAT ACA TCA AAC AGG CGC CTC TG), and inserted into the lentivirus vector to generate the gK_wt_ expressing vector pCDHpuro-gK_wt_. Then, the *gK* gene was mutated (A40V, C82S, M223I, L224V, V309M) to generate gK_mut_ expressing vector pCDHpuro-gK_mut_. The lentiviruses were packaged in human embryonic kidney cell line HEK293T (ATCC, #CRL- 11268) as described previously [24], and used to transduce Vero cells. The transduced cells were selected with 4 μg/ml puromycin (Sigma) for one week, and then maintained under the selection pressure of 2 μg/ml puromycin. The resulted cell lines were designated as Vero-gK_wt_ and Vero-gK_mut_, respectively. All these cells were cultured with Dulbecco’s modified Eagle medium (DMEM, Gibco/Life Technologies) containing 10% fetal bovine serum (FBS, Gibco/Life Technologies) and penicillin (100 U/ml)-streptomycin (100 μg/ml) (Gibco/Life Technologies).

Fetal mouse cortical neurons were isolated and cultured as described previously [25, 26]. Briefly, the cerebral cortex was dissected from the forebrain of C57BL/6 mouse fetuses at embryonic day 18.5 (E18.5), and then dissociated with 0.25% trypsin (Gibco/Life Technologies)/DNase I (Sigma) for 15 min at 37 °C. After being washed with Ca^2+^/Mg^2+^ free Hank’s Balanced Salt Solution (HBSS) (Gibco/Life Technologies), the isolated neurons were resuspended, plated in microfluidic plates, and cultured in Neurobasal medium (Gibco/Life Technologies) supplemented with B27 (2%) (Gibco/Life Technologies), GlutaMAX (25 μM) (Gibco/Life Technologies) and penicillin (100 U/ml)-streptomycin (100 μg/ml). The medium was refreshed every 2 days.

All cells were cultured at 37 °C in a humidified atmosphere containing 5% CO_2_.

### Construction and propagation of the recombinant viruses: H129-dgK-G4, H129-dTK-G4, and AAV helpers

**Fig. 1:**
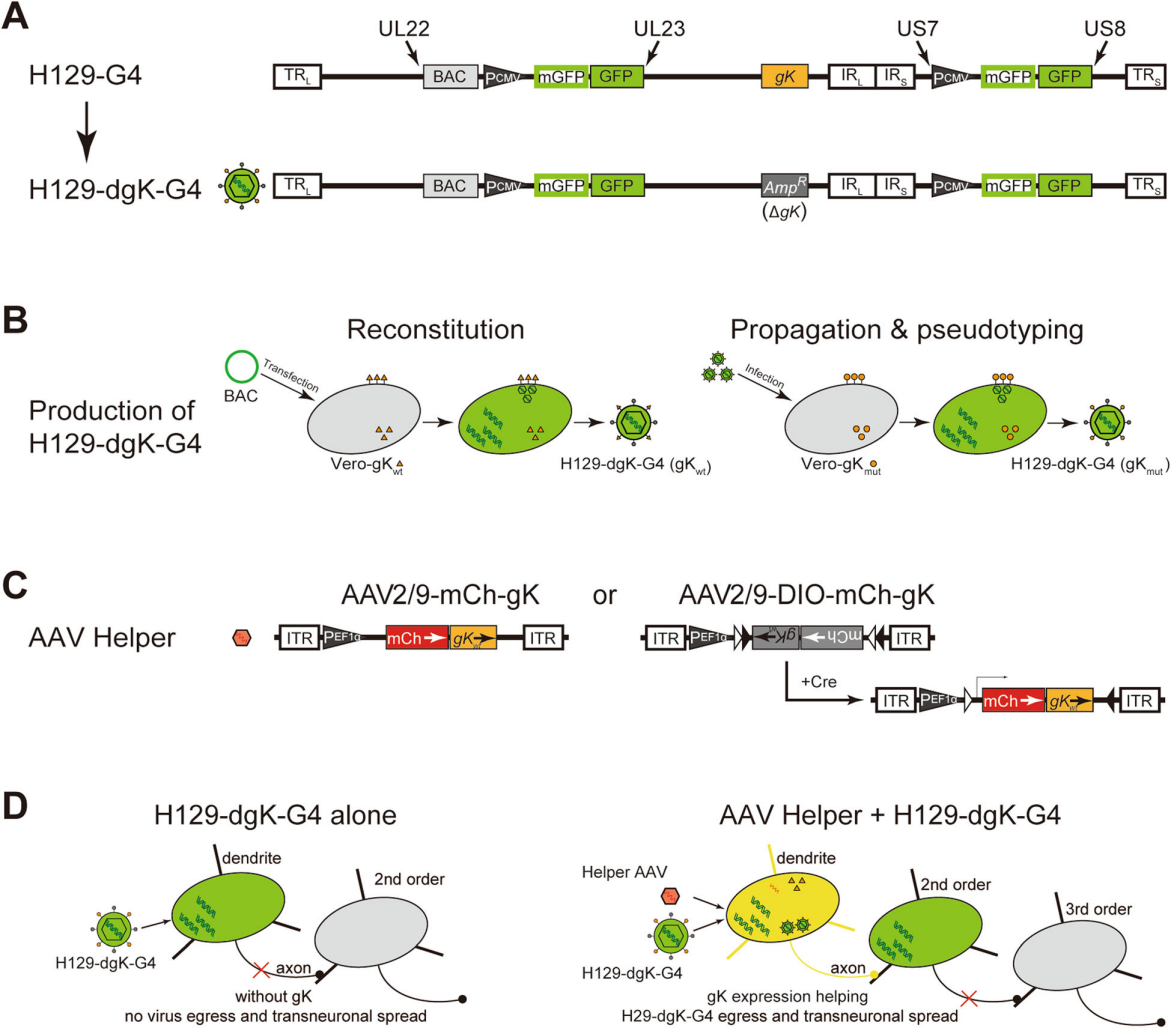
H129-dgK-G4: construction, production, and illustration of anterograde monosynaptic tracing. **A**. Schematic genome structure of H129-dgK-G4. Based on the previously created H129-G4, H129-dgK-G4 was generated by knocking out the *gK* gene and replacing it with the ampicillin resistance gene (*Amp*^*R*^). **B.** Reconstitution and propagation of H129-dgK-G4. The BAC_H129-dgK-G4_ DNA was transfected into Vero cells stably expressing wildtype gK (indicated with the orange triangle) (Vero-gK_wt_) to reconstitute the recombinant virus with wildtype gK residing on the viral envelope, designated as H129-dgK-G4(gK_wt_) (left panel). As the seed virus, H129-dgK-G4(gK_wt_) was further propagated in Vero cells stably expressing gK_mut_ (Vero-gK_mut_). The obtained recombinant virus is pseudotyped with the mutant gK (gK_mut_, indicated with the orange circle), and designated as H129-dgK-G4(gK_mut_) (right panel). To simplify the labeling, H129-dgK-G4 represents the H129-dgK-G4(gK_mut_), unless specified. **C**. Schematic genome structure of the AAV helpers for H129-dgK-G4 monosynaptic tracing. AAV2/9 expressing mCherry and gK_wt_ is used as the helper virus for monosynaptic tracing. To simplify the labeling, the gK in AAVs all indicates gK_wt_. AAV2/9-mCh-gK expresses gK and mCherry constitutively under the control of EF1α promoter, while AAV2/9-DIO-mCh-gK expresses them only in the presence of Cre recombinase. **D.** Schematic mechanism of H129-dgK-G4 monosynaptic tracing. After infecting the starter neurons, H129-dgK-G4 replicates its genome and synthesizes viral proteins as well as the fluorescent genes, and thus brightly labels the neurons. The deficiency of gK impairs viral egress and transmission (left panel). Coinfected AAV helper expresses gK *in trans* and assists the egress of H129-dgK-G4, which in turn anterogradely transmits to and labels the post-synaptic neurons (right panel). Then, H129-dgK-G4 is restrained in the 2nd order neurons, and does not further spread due to the lack of the AAV helper

H129-dgK-G4 was derived from the previously introduced H129-G4, which contains 2 tandem GFP expression cassettes, each with one GFP and one membrane-bound GFP (mGFP) coding gene [10, 12]. Using the bacterial artificial chromosome (BAC) and homologous recombination technique, the *gK* gene (*UL53*) was knocked out by replacing it with an Ampicillin resistant gene (*Amp*^*R*^) (Fig. 1A) [10]. The obtained recombinant BAC DNA was transfected into Vero-gK_wt_ to reconstitute the recombinant virus coated with gK_wt_, labeled as H129-dgK-G4(gK_wt_) (Fig. 1B, left panel). The reconstituted virus was further propagated in Vero-gK_mut_ to generate the gK_mut_ pseudotyped recombinant virus H129-dgK-G4(gK_mut_), which is further applied as the novel anterograde monosynaptic tracer (Fig. 1B, right panel). To simplify the labeling, all H129-dgK-G4 in the manuscript represent the gK_mut_ pseudotyped virus H129-dgK-G4(gK_mut_), unless specifically indicated. The H129-derived tracer was propagated following our previously published protocol [13], and the ready-to-use H129-dgK-G4 typically reaches an average titer of 5 × 10^8^ pfu/ml.

For monosynaptic tracing, the AAV helpers of AAV2/9-mCh-gK and AAV2/9-DIO-mCh-gK were constructed and packaged as described previously, which express P2A linked mCherry and gK_wt_ either constitutively or via a Cre-dependent manner, respectively (Fig. 1C) [10, 13].

Similarly, H129-dTK-G4 and the corresponding AAV helper (AAV2/9-TK-mCh) were generated and propagated/packaged as described previously [10, 13].

### Microfluidic assay

The microfluidic plate has been introduced previously and was fabricated following the described protocol [10, 26]. In brief, it contains two isolated chambers connected by multiple microchannels (700 μm long, 10 μm wide, and 3 μm deep), which allows only the axons to grow through but not the somas or dendrites. Quality control of the microfluidic plates was performed using 5 randomly selected plates from each fabrication batch (50 plates) to examine the potential inter-chamber leakage. Vero cells (5 × 10^5^) were cultured in one chamber (day 1), where cells cannot grow through the microchannels to reach the opposite chamber, and H129-G4 (1 × 10^6^ pfu) was added to the opposite chamber on day 5. To avoid virus diffusion, less medium volume was maintained in the virus-inoculating chamber to achieve lower hydrostatic pressure. On day 8, the plates were examined for GFP signal caused by H129-G4 infection of Vero cells, which indicates possibly inter-chamber leakage. Then the certified batch of microfluidic plates was applied for further experiments only when no leakage occurred in any tested plates.

For microfluidic assays, fresh isolated fetal mouse cortical neurons (1 × 10^6^) were plated into both chambers (on day 1 and day 5 respectively) or one chamber (on day 1) of the microfluidic plate. The viruses were added into the indicated chamber with lower hydrostatic pressure. To test the transneuronal transmission, AAV2/9-mCh-gK (8 × 10^9^ vg, when indicated) were added to the efferent chambers on day 14, and H129-derived tracers (1 × 10^6^ pfu, as indicated) were added to the same chamber on day 20. GFP-positive neurons were examined 2 days after adding the H129-derived tracers. To test the retrograde labeling caused by axon terminal invasion, H129-derived tracers (1 × 10^6^ pfu) were added to the axon terminal chamber on day 14. GFP-positive neurons were examined 1 day after adding the H129-derived tracers.

### Intracranial injection of the viral tracers

Wildtype C57BL/6 mice were purchased from Beijing Vital River Laboratory Animal Technology company. GAD2-Cre transgenic mice, which specifically express Cre recombinase under the control of glutamic acid decarboxylase promoter, were provided by the Laboratory Animal Resource Center at the Chinese Institute for Brain Research, Beijing (CIBR), and the 3 × Tg-AD mice showing symptoms of Alzheimer’s disease were provided by the Department of Physiology, School of Basic Medicine and Tongji Medical College, Huazhong University of Science and Technology. The parameters of the mouse brain regions were determined according to the Mouse Brain Atlas by the mediolateral (ML), anteroposterior (AP) and dorsoventral (DV) distances to Bregma [27]. The indicated viral tracers were intracranially administrated into the target brain region using a motorized stereotaxic injector (Stoelting) under anesthesia.

AAV helpers (1.0 × 10^12^ vg/ml, 100–150 nl) and H129-derived tracers (5.0 × 10^8^ pfu/ml, 100–150 nl) were sequentially injected into the same location of the indicated brain regions on day 1 and 22, respectively. On day 27, animals were anesthetized and perfused with sterile normal saline and 4% paraformaldehyde (PFA) solution, and the whole brain was carefully collected. The obtained brains were fixed with 4% PFA, dehydrated in 30% sucrose, and stored at 4 °C for further cryosection and imaging. When indicated, the same amount of H129-derived tracers were injected into the indicated brain regions alone, and the brains were collected at 5 days post-injection.

### Cryosection and imaging

After fixation and dehydration, the obtained brains were coronally cryo-sectioned to 40 μm-thick slices using a microtome (HM550, Thermo / Life Technologies). The only staining is to show cell nuclei by counterstaining with DAPI (Cat. #10236276001, Roche), and all the GFP signals are natural origins without any signal amplification. All images were obtained using a Nikon’s A1R MP+ confocal microscope equipped with a fast high-resolution galvanometer scanner.

To count the labeled neuron number in the indicated brain regions of each mouse, the coronal brain slices at similar positions were observed with an interval of 160 μm (one from continuous 5 40 μm-thick slices), and the labeled neurons at the indicated brain regions were counted. To measure the labeling intensity of GFP positive neurons, three slices at similar positions from the indicated brain regions of the same brain were selected, and the GFP intensity was quantified by ImageJ software v1.60 (NIH, USA). By this method, the quantified value of mean GFP intensity of the labeled cells was shown as the arbitrary unit (AU), which was calculated as IntDen/Area (IntDen, Integrated Density of GFP-labeled neurons; area, the total area of GFP-labeled neurons).

### Statistical analysis

Each experiment was performed in triplicate, and the results were presented as means ± SEM (Standard Error of the Mean) from at least three independent experiments or animals. Appropriate statistical tests were applied in the data analysis, including Student’s t-test or linear mixed-effect model (LME) analysis. LME has been widely used to analyze correlated data such as clustered data [28, 29]. Differences were considered to be significant when the *p-*value was < 0.05.

## Results

### Construction of H129-dgK-G4

Using the bacterial artificial chromosome (BAC) technique and homologous recombination method, we knocked out the *gK* gene from H129-G4, the previously introduced polysynaptic tracer, by replacing it with the ampicillin-resistant gene (*Amp*^*R*^) (Fig. 1A) [10, 12]. The generated BAC DNA was transfected into Vero cells stably expressing wildtype gK (gK_wt_) (Vero-gK_wt_) to reconstitute the recombinant virus H129-dgK-G4 (gK_wt_) (Fig. 1B, left panel). gK is associated with HSV-1 retrograde infection through axon invasion and axonal transport to the soma, and the recombinant HSV-1 coated with the gK protein from strain KOS displayed a dramatic decrease of retrograde infection efficiency through the axons than that coated with the gK from strain McKrae [23]. We generated the Vero cell line stably expressing the mutant gK (gK_mut_, A40V, C82S, M223I, L224V, V309M), namely Vero-gK_mut_. In Vero-gK_mut_, the novel anterograde monosynaptic tracer H129-dgK-G4(gK_mut_) was produced by using either the reconstituted H129-dgK-G4 (gK_wt_) or H129-dgK-G4(gK_mut_) as a seed virus. The novel tracer H129-dgK-G4(gK_mut_) is deficient with *gK* in the genome and is pseudotyped with gK_mut_ on the envelope (Fig. 1B, right panel). All the H129-dgK-G4 tracer used in the present study was propagated in the Vero-gK_mut_ cells and pseudotyped by gK_mut_, unless specified.

The deficiency of gK does not influence viral genome replication, viral protein synthesis, and primary viral assembly, but severely impairs viral egress and transmission [23], which not only hinders virus yield dramatically but also blocks their transmitting/labeling neurons in both anterograde and retrograde ways. Thus, appropriate AAV helpers complementarily expressing gK are required to further assist the monosynaptic transmission of H129-dgK-G4. We constructed and packaged AAV helpers, namely AAV2/9-mCh-gK and AAV2/9-DIO-mCh-gK, which bicistronically express mCherry and gK_wt_ with a P2A linker constructively or in a Cre-recombinase dependent manner, respectively (Fig. 1C). When infecting neurons, H129-dgK-G4 replicates its genome, synthesizes viral proteins, and simultaneously expresses abundant mGFP/GFP so as to label the infected neurons with strong fluorescence intensity. However, gK deficiency abolishes viral egress, transneuronal transmission, and infection of H129-dgK-G4 (Fig. 1D, left panel). When infecting the same neuron, AAV helper compensatorily expresses gK_wt_ protein that resides on the envelope of H129-dgK-G4 virion. Then the gK_wt_ compensated H129-dgK-G4 can egress and transmit one step down to the postsynaptic neurons, which are then labeled by mGFP/GFP expressed in situ (Fig. 1D, right panel).

Upon virus entry, gK_wt_ plays an important role in axon terminal invasion while gK_mut_ limits axon terminal invasion in vitro [23]. In the initial injection site, high concentration of tracer is usually applied to achieve efficient tracing, thus gK_mut_ pseudotyping may potentially contribute to avoiding or limiting the axon terminal invasion of H129-dgK-G4. Once H129 enters the neurons and replicates in them, their progeny virions released from the infected neurons have only a restricted probability to invade new neurons via axon terminal invasion, therefore it is not necessary to use gK_mut_ again to complement the replication of H129-dgK-G4. Instead, the origin gK_wt_ of H129, which displays higher efficiency to assist H129-dgK-G4 spreading [23], is more helpful to serve as the compensating viral protein. Taking together, to limit axon terminal invasion, the gK_mut_ pseudotyped H129-dgK-G4 is designed as the tracer, and then after virus entry and replication, gK_wt_ expressed by the AAV helper assists the progeny virions anterograde axonal transport and spread with higher efficiency. Thus AAV helper compensatorily expresses gK_wt_ is designed to assist the monosynaptic tracing.

### Anterograde monosynaptic labeling of H129-dgK-G4

**Fig. 2:**
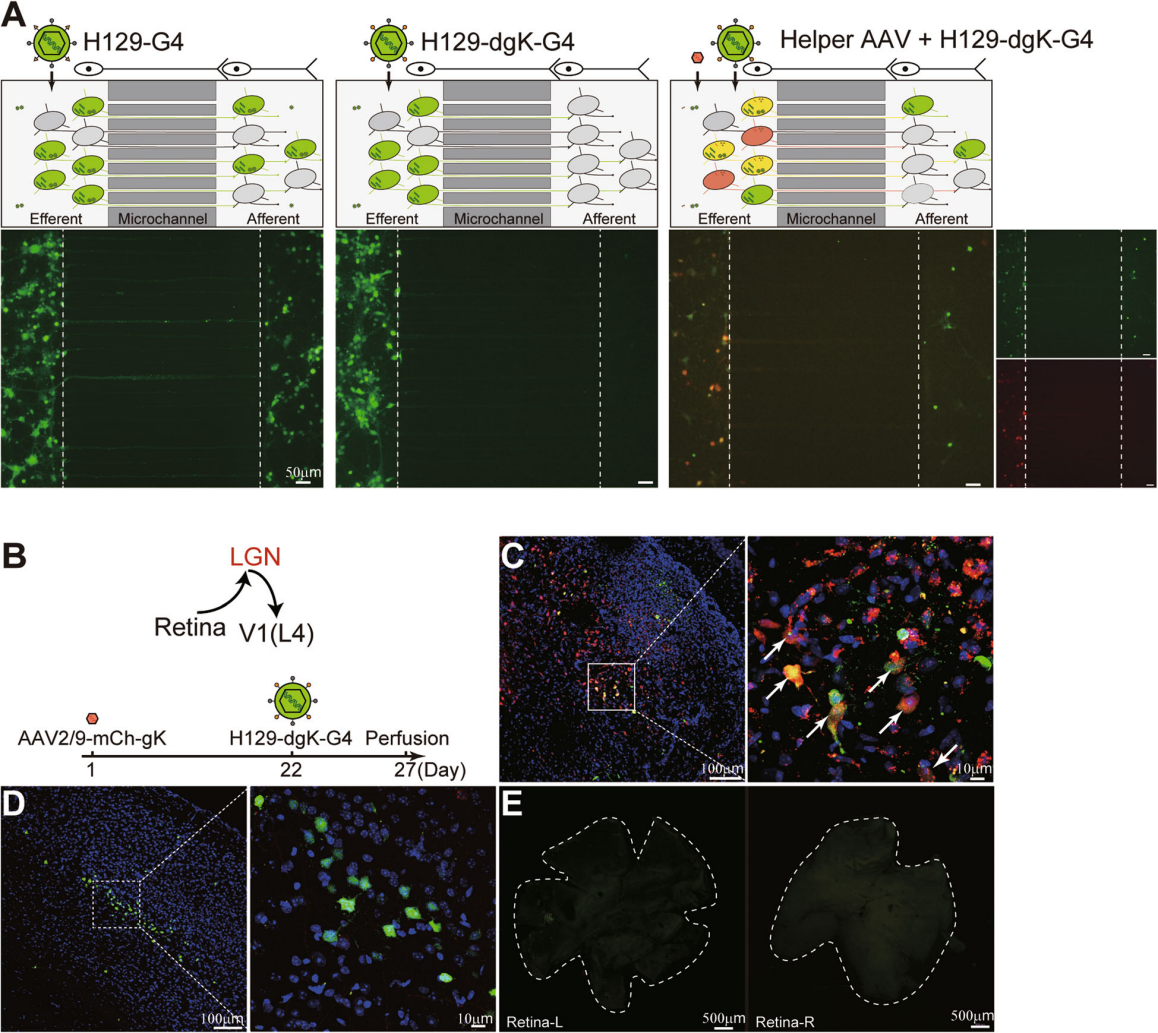
In vitro and in vivo anterograde monosynaptic labeling by H129-dgK-G4 and its AAV helper*.* **A.** In vitro transneuronal labeling of H129-dgK-G4. 1 × 10^6^ fetal mouse cortical neurons were sequentially plated in both chambers of the microfluidic plates at day 1 and day 5, and cultured for additional 14 days to allow synapse formation in the afferent chamber. The indicated viruses were inoculated to the efferent side with the dosage of 1 × 10^6^ pfu for H129-derived tracers and 8 × 10^9^ vg for AAV2/9-mCh-gK. The labeled neurons in the afferent chamber were examined at 2 days post infection (dpi). The dotted lines indicate the borders between chambers and the microchannels, and shown are the representative images from 6 microfluidic plates per group performed in 3 independent experiments. **B–E.** In vivo anterograde monosynaptic labeling of H129-dgK-G4 with the helper AAV2/9-mCh-gK. In the simplified visual circuit pathway (**B**, upper panel), neurons in the lateral geniculate nucleus (LGN) receive the projection from the retina, and innervate the neurons in layer 4 of the primary visual cortex (V1(L4)). AAV2/9-mCh-gK (1.0 × 10^12^ vg/ml, 100 nl) and H129-dgK-G4 (5.0 × 10^8^ pfu/ml, 100 nl) were sequentially injected into LGN at day 1 and day 22, and the brains were collected at day 27 after perfusion (**B**, lower panel). Shown are the representative images of the injection site (**C**), V1 (**D**), and retinas (**E**) from the same mouse. The white arrow indicates the cells colabeled by mCherry and GFP, representing the potential tracing starter neurons

To characterize the transmitting and labeling property of H129-dgK-G4, neurons were cultured in the microfluidic plates as described previously [10, 13, 26, 30] and in the Materials and Methods. The same dosage of H129-derived tracers of H129-G4 or H129-dgK-G4 (1 × 10^6^ pfu) was inoculated to infect the neurons in the efferent chamber (Fig. 2A). Consistent with previously published results [10], the polysynaptic tracer H129-G4 labeled neurons in both chambers, indicating it replicated in the efferent neurons, then transmitted to the downstream afferent neurons through the axons in microchannels, and labeled the downstream neurons (Fig. 2A, left panel). H129-dgK-G4 alone well labeled the efferent neurons, but failed to transmit to and label the downstream afferent neurons (Fig. 2A, middle panel). To express high enough gK to assist H129-dgK-G4 replicating/ transmitting to downstream neurons but avoid AAV2/9-mCh-gK transmitting by itself, the condition of AAV2/9-mCh-gK was carefully evaluated. A few afferent neurons were labeled by AAV at the administration dosage of 1 × 10^10^ vg. At the dosage of or less than 8 × 10^9^ vg, AAV2/9-mCh-gK did not transmit to and label afferent neurons, but successfully assisted H129-dgK-G4 anterograde transmission in a dose-dependent manner (Additional file: Fig. S1). So 8 × 10^9^ vg was then chosen as the optimized AAV administration dose in the microfluidic assay in further experiments. When AAV2/9-mCh-gK (8 × 10^9^ vg) was administrated 7 days prior to the H129-dgK-G4 inoculation, AAV2/9-mCh-gK and H129-dgK-G4 coinfection labeled many efferent neurons with mGFP/GFP and mCherry (merged as yellow). Some of the downstream afferent neurons were clearly labeled with GFP, suggesting H129-dgK-G4 anterogradely transmitted to the postsynaptic neurons with the AAV helper compensatorily providing gK (Fig. 2A, right panel).

Similar to the in vitro result obtained in the microfluidic plates, H129-dgK-G4 also displayed the capability of anterograde monosynaptic tracing in vivo stringently depending on AAV helper compensating gK. When applied alone, H129-dgK-G4 only infected and labeled neurons around the injection site, but did not spread from the tested brain regions, including the primary motor cortex (M1), auditory cortex (Au), and dentate gyrus (DG) of wildtype C57BL/6 mice (Additional file: Fig. S2). However, upon the presence of AAV helper (AAV2/9-mCh-gK), H129-dgK-G4 achieved anterograde monosynaptic tracing in vivo, which is demonstrated by the unidirectional pathway retina-LGN-V1(L4). As shown in Fig. 2B, AAV2/9-mCh-gK (1.0 × 10^12^ vg/ml, 100 nl) and H129-dgK-G4 (5.0 × 10^8^ pfu/ml, 100 nl) were sequentially injected into the lateral geniculate nucleus (LGN, AP: − 2.30 mm; ML: − 2.13 mm; DV: − 2.75 mm) of wildtype C57BL/6 mice on day 1 and day 22, respectively, and brains were collected on day 27. Neurons expressing both mCherry and GFP (merged as yellow) were observed around the injection site LGN (Fig. 2C, indicated with the white arrow), representing the potential starter neurons for initial transmission. In the downstream brain region V1, GFP-labeled neurons were only observed in Layer IV (L4), which is directly innervated by LGN [31], indicating that only H129-dgK-G4 anterogradely transmits through one order to the postsynaptic neurons with the AAV helper compensating gK (Fig. 2D), but not the AAV helper. H129 has been shown to have potential retrograde transmission [15]. No GFP positive neuron in the retina, a direct upstream region of the LGN, was observed, suggesting there is no retrograde transmission and labeling of H129-dgK-G4 in the experimental condition (Fig. 2E). Moreover, no neurons were observed at other areas beyond the brain regions directly innervated by LGN (data not shown), indicating the anterograde transneuronal tracing of H129-dgK-G4 is monosynaptic.

Altogether, these data suggest that H129-dgK-G4 can transmit to and label downstream neurons in an anterograde monosynaptic manner, which is dependent on AAV helper compensating gK.

### Strong labeling intensity and high tracing efficiency of H129-dgK-G4

**Fig. 3:**
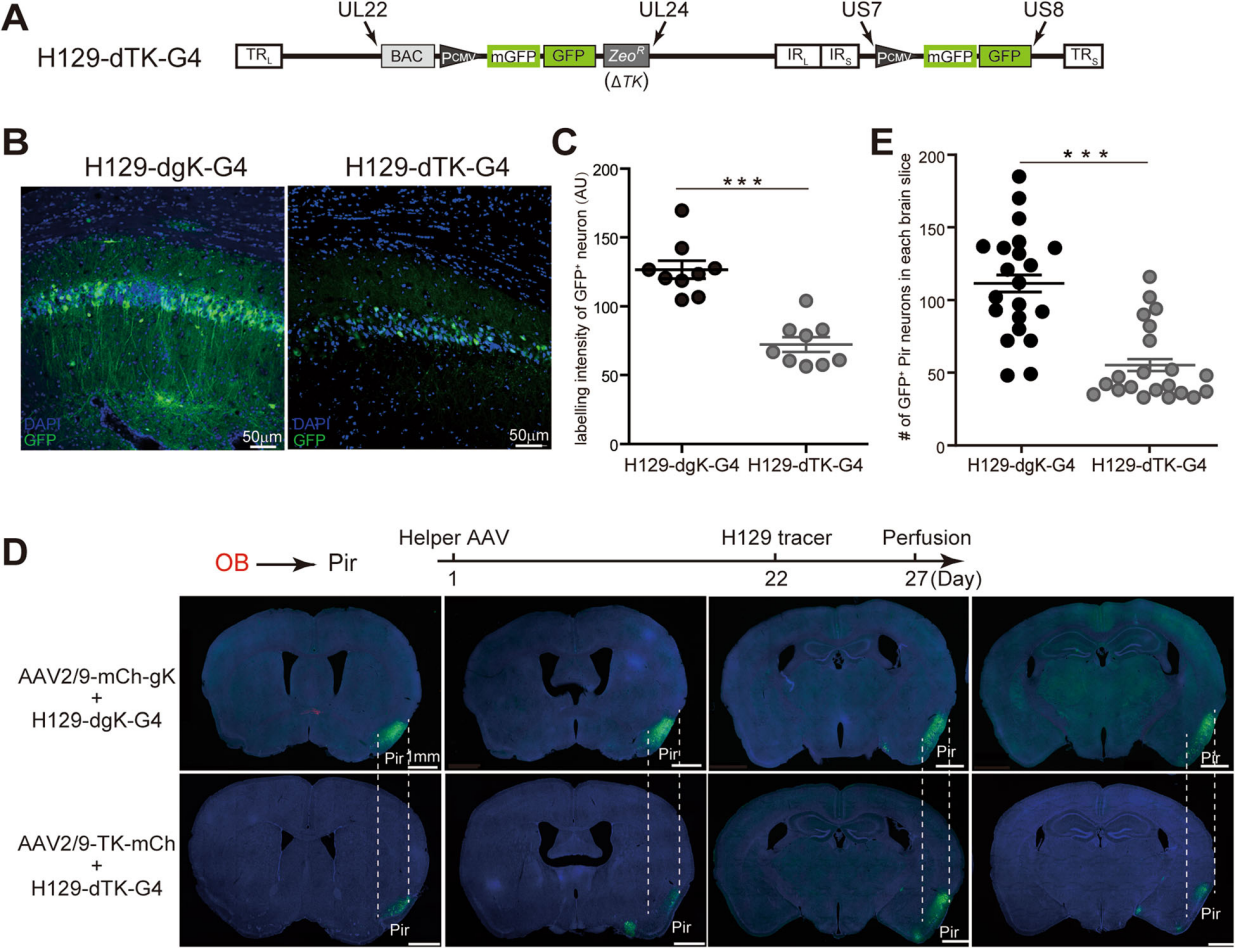
Stronger labeling intensity and higher tracing efficiency of H129-dgK-G4. **A**. Schematic genome structure of H129-dTK-G4. **B-C**. Comparison of labeling intensity between H129-dgK-G4 and H129-dTK-G4. H129-dgK-G4 and H129-dTK-G4 alone were injected into the CA1 of wildtype C57BL/6 mice with the same dosage (5.0 × 10^8^ pfu/ml, 100 nl) respectively, and the brains were collected one day later. The representative images from 3 mice of each group are shown (**B**). The labeling intensity of the GFP^+^ neurons was measured and analyzed by Image J software, and quantitated as the average brightness of the labeled neurons (AU, arbitrary unit). 3 position-matched slices from each mouse and 3 mice per group were applied for the analysis. Statistical significance was analyzed by Student’s t-test. ***, *p* < 0.001(C). **D-E**. Comparison of the transneuronal labeling efficiency between H129-dgK-G4 and H129-dTK-G4. The appropriate AAV helper virus (1.0 × 10^12^ vg/ml, 150 nl) and H129-derived monosynaptic tracer (5.0 × 10^8^ pfu/ml, 150 nl) of H129-dgK-G4 or H129-dTK-G4 were sequentially injected into the olfactory bulb (OB) of wildtype C57BL/6 mice at day 1 and day 22, and the brains were perfused and collected at day 27. The representative images of the projecting region of the piriform cortex (Pir) from 3 mice of each group are shown (**D**). The number of GFP-labeled neurons in Pir of each position-matched brain slice was counted. Statistical significance was determined by linear mixed-effect model (LME) analysis. ***, *p* < 0.001 (**E**)

We have previously introduced H129-dTK-tdT, the first anterograde monosynaptic tracer derived from H129, and then an updated version H129-dTK-T2 with an improved labeling intensity by adding an extra tdTomato expression cassette [10, 13]. We also generated H129-dTK-G4 (Fig. 3A), whose labeling intensity is similar to H129-dTK-T2. The strategy of generating these monosynaptic tracers is dependent on TK deficiency. TK deficiency impairs viral genome replication in neurons, and limits viral protein synthesis as well as the fluorescent protein reporters. Therefore, all the TK deficient tracers share the inherent drawback of low labeling intensity in the postsynaptic neurons [12], which limits their transsynaptic tracing applications.

In the present study, the structural gene *gK* was knocked out instead of *TK*. H129-dgK-G4 and H129-dTK-G4 were both derived from H129-G4, only differently deficient with gK or TK, respectively. The labeling intensity and tracing efficiency were quantitated and compared between H129-dgK-G4 and H129-dTK-G4. The mechanism for monosynaptic tracing of H129-dgK-G4 or H129-dTK-G4 along with the AAV helper, only the H129-derived deficient tracers, but not the AAV helpers, transmit to and label the postsynaptic neurons. Thus, to mimic the similar condition, we injected H129-dgK-G4 or H129-dTK-G4 alone into the CA1 (AP: − 2.18 mm; ML: − 1.00 mm; DV: − 1.50 mm) of wildtype C57BL/6 mice with the same dosage (5 × 10^8^ pfu/ml, 100 nl). As shown in the representative images (the original GFP signal and DAPI counterstaining), H129-dgK-G4 labeled neurons around the injection site with stronger labeling intensity than H129-dTK-G4 (Fig. 3B). Quantitation analysis on the fluorescence brightness of the labeled neurons showed that the average labeling intensity of H129-dgK-G4 is 1.76-fold higher than that of H129-dTK-G4 (127 ± 7 AU vs 72 ± 5 AU) (Fig. 3C). Therefore, H129-dgK-G4 has stronger labeling intensity.

The increased labeling intensity of H129-dgK-G4 further improves the tracing efficiency by visualizing more labeled postsynaptic neurons. To assess the tracing efficiency, H129-dgK-G4 and H129-dTK-G4 were applied alongside the AAV helper in mapping the olfactory pathways, and the labeled postsynaptic neurons were counted and analyzed. H129-dgK-G4 or H129-dTK-G4 (5.0 × 10^8^ pfu/ml, 150 nl) was injected into the olfactory bulb (OB, AP: + 4.28 mm; ML: − 0.50 mm; DV: − 2.50 mm) of wildtype C57BL/6 mice along with the corresponding AAV helper, AAV2/9-mCh-gK or AAV2/9-TK-mCh (1.0 × 10^12^ vg/ml, 150 nl), respectively. Both tracers anterogradely transmitted to the downstream brain regions and labeled the neurons, represented by the piriform cortex (Pir) (Fig. 3D). We counted GFP-labeled (GFP^+^) Pir neurons in the position-matched brain slices of each mouse brain (7 slices per mouse, and 3 mice per group). An average of 112 ± 11 GFP^+^ neurons was observed in the Pir of each slice by H129-dgK-G4 tracing, while only half amount of GFP^+^ Pir neurons (56 ± 13) was observed by H129-dTK-G4 tracing (Fig. 3E). Compared to H129-dTK-G4, H129-dgK-G4 has doubled (2.00-fold) tracing efficiency, which is an extra bonus of the new tracer.

Taken together, H129-dgK-G4 achieves a significant increase in both the labeling intensity and tracing efficiency, which is a great improvement of the gK deficient anterograde mono-synaptic H129 tracer.

### Significantly reduced retrograde labeling of gK_mut_ pseudotyped H129-dgK-G4

H129 may invade through the axon terminal, which makes the neurons in the upstream regions be retrogradely labeled by H129-derived anterograde tracers [15, 16]. Our previous study showed that the retrograde labeling ratio of the H129-derived tracer is associated with the brain regions, viral titer, administration dosage, and the tracing time [10, 12]. Although carefully optimizing these experimental parameters can limit the potential retrograde labeling, a fundamental solution is still required to overcome the natural viral property of axon terminal invasion, so that to further minimize potential retrograde labeling for a higher tracing specificity.

The amino acid sequences of gK protein are slightly different between different HSV-1 strains, which is associated with viral axon terminal invasion efficiency [23]. We hypothesized that pseudotyping H129-dgK-G4 with the mutant gK protein might limit viral axon terminal invasion and thus reduce the resulting retrograde labeling probability. Therefore, the Vero cell line stably expressing the mutant gK (gK_mut_, A40V, C82S, M223I, L224V, V309M) was generated, namely Vero-gK_mut_, and used to propagate the gK_mut_ pseudotyped H129-dgK-G4.

**Fig. 4:**
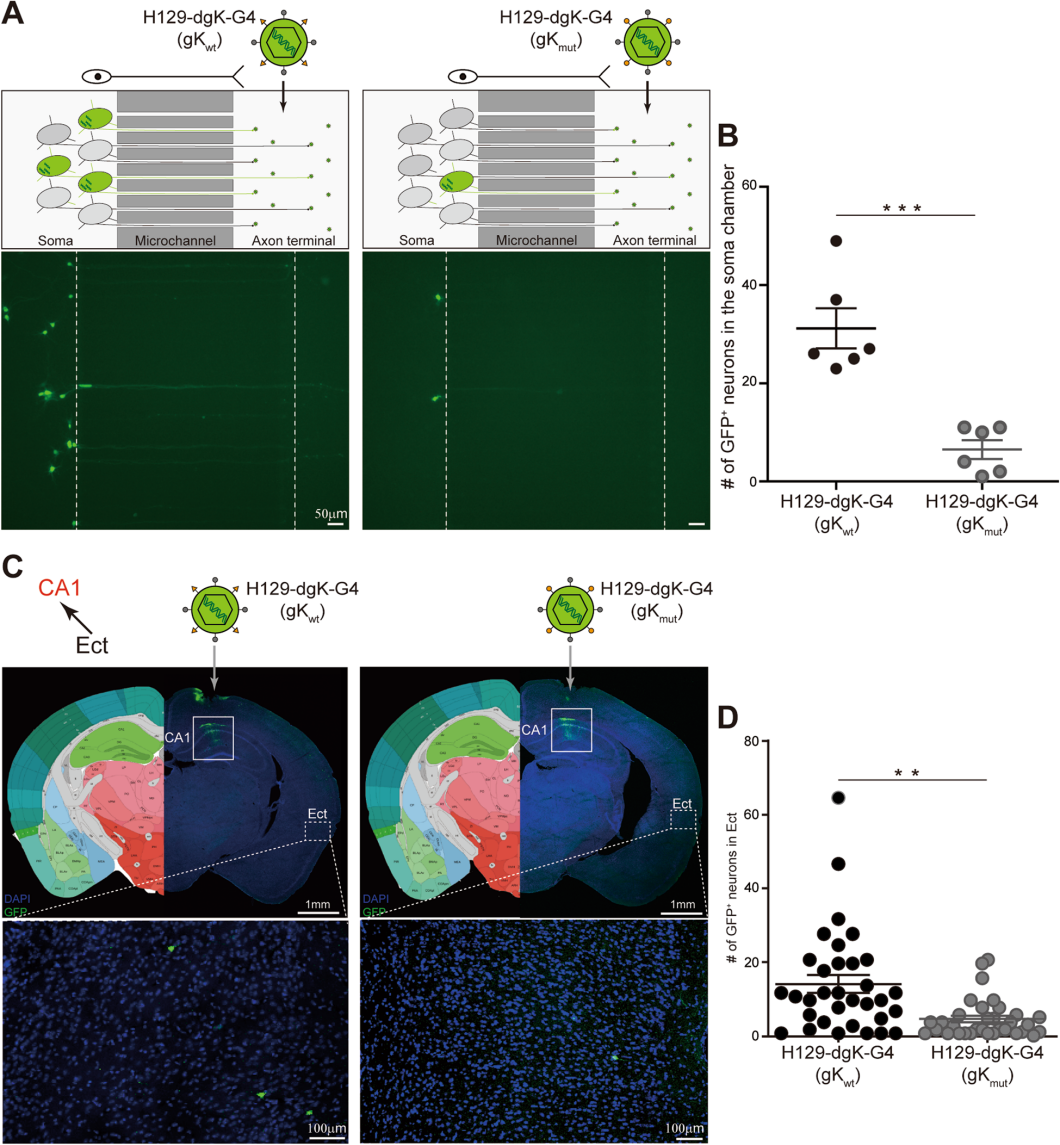
Significantly reduced retrograde labeling of gK_mut_ pseudotyped H129-dgK-G4*.* **A**, **B**. In vitro comparison of retrograde labeling by axon terminal invasion between H129-dgK-G4(gK_wt_) and H129-dgK-G4(gK_mut_). 1 × 10^6^ fetal mouse cortical neurons were seeded into the soma chamber of the microfluidic plates, and cultured for 14 days to allow axons to grow through the microchannel and reach the contralateral chamber. Then, 1 × 10^6^ pfu of H129-dgK-G4(gK_wt_) or H129-dgK-G4(gK_mut_) was added to the axon terminal side. GFP-labeled neurons were monitored and counted at 1 dpi. Shown are the representative images from 6 microchannel plates of each group performed in 2 independent experiments (**A**). The GFP positive neurons were counted in each chamber, and the statistical significance was analyzed by Student’s t-test. ***, *p* < 0.001 (**B**). **C**, **D**. In vivo comparison of retrogradely labeling between H129-dgK-G4(gK_wt_) and H129-dgK-G4(gK_mut_). H129-dgK-G4(gK_wt_) or H129-dgK-G4(gK_mut_) was injected into CA1 (indicated with the solid-line box) of wildtype C57BL/6 mice with the same dosage (5.0 × 10^8^ pfu/ml, 100 nl), and the brains were collected at 1 dpi. The numbers of the GFP-labeled neurons at the ectorhinal cortex (Ect), an upstream nucleus of CA1, were examined and counted in each brain. Shown are the representative images from 3 mice of each group (**C**, upper panel), and the magnified images of Ect (indicated with the dashed-line box) (**C**, bottom panel). The left hemisphere displays the corresponding sections from Allen Brain Reference Atlases (Image credit: Allen Institute). Numbers of the GFP^+^ neuron in the position-matched brain slices were counted (11 slices/mouse × 3 mice), and statistical significance was analyzed by LME. **, *p* < 0.01 (**D**)

The retrograde labeling ratio was quantitated, and comparison was performed between H129-dgK-G4 pseudotyped with gK_mut_ (H129-dgK-G4(gK_mut_)) and that coated with gK_wt_ (H129-dgK-G4(gK_wt_)) under the same conditions. In vitro, 1 × 10^6^ neurons were cultured in one chamber (soma chamber) of each microfluidic plate, and the axons grew through the microchannels reaching the contralateral chamber (axon terminal chamber). The same dosage (1 × 10^6^ pfu) of H129-dgK-G4(gK_mut_) or H129-dgK-G4(gK_wt_) was added to the axon terminal chamber. At 1 day post infection (dpi), GFP-labeled neurons, caused by viral tracer infection via axon terminal invasion, were monitored and counted in the soma chambers (Fig. 4A). An average of 31 ± 4 neurons in the soma chambers were retrogradely labeled by H129-dgK-G4(gK_wt_), while only 7 ± 2 by H129-dgK-G4(gK_mut_). Therefore, gK_mut_ pseudotyping dramatically reduced the retrograde labeling of H129-dgK-G4 tracer by 77% in vitro (Fig. 4B).

Similarly, gK_mut_ pseudotyping decreased the retrograde labeling In vivo. H129-dgK-G4(gK_mut_) or H129-dgK-G4(gK_wt_) (5.0 × 10^8^ pfu/ml, 100 nl) was injected into the CA1 (AP: − 2.18 mm; ML: − 1.00 mm; DV: − 1.50 mm) of wildtype C57BL/6 mice. Both tracers nicely labeled the neurons around the injection site (Fig. 4C, upper panel). A few GFP positive neurons were observed in the ectorhinal cortex (Ect), which is an upstream brain region innervating CA1, indicating the retrograde labeling by virus via axon terminal invasion from CA1 (Fig. 4C, lower panel). The GFP-labeled Ect neurons were carefully counted in the position-matched brain slices from each mouse. It is shown that H129-dgK-G4(gK_wt_) averagely labeled 13 ± 2 Ect neurons in each brain slice, while only 3 ± 3 were labeled by H129-dgK-G4(gK_mut_) (Fig. 4D). Thus, gK_mut_ pseudotyping the H129-dgK-G4 reduced the retrograde labeling by 77%.

Notably, retrograde labeling of H129-dgK-G4(gK_mut_) was only observed from CA1, but not from any other tested brain regions, including olfactory bulb, primary motor cortex, infralimbic cortex, dentate gyrus, Auditory cortex, primary visual cortex, and median raphe nucleus (data not shown). And low level of retrograde labeling from CA1 occurred only at a high injection dosage (5.0 × 10^8^ pfu/ml, 100 nl) of H129-dgK-G4(gK_mut_). When gK_mut_ pseudotyped H129-dgK-G4 was injected at any lower dosages (1.0 × 10^8^ or 2.5 × 10^8^ pfu/ml, 100 nl) or other tested brain regions, no retrograde labeling was observed (data not shown).

Taken together, both the in vitro and in vivo assessments confirm that gK_mut_ pseudotyping reduces the retrograde labeling incidence of H129-dgK-G4. It represents a more anterograde-specific monosynaptic tracer with less retrograde labeling, and limits potential misleading interpret. Thus, all H129-dgK-G4 used throughout the manuscript were propagated in Vero-gK_mut_ cell and is gK_mut_ pseudotyped H129-dgK-G4 tracer.

### Anterograde monosynaptic tracing of H129-dgK-G4

**Fig. 5:**
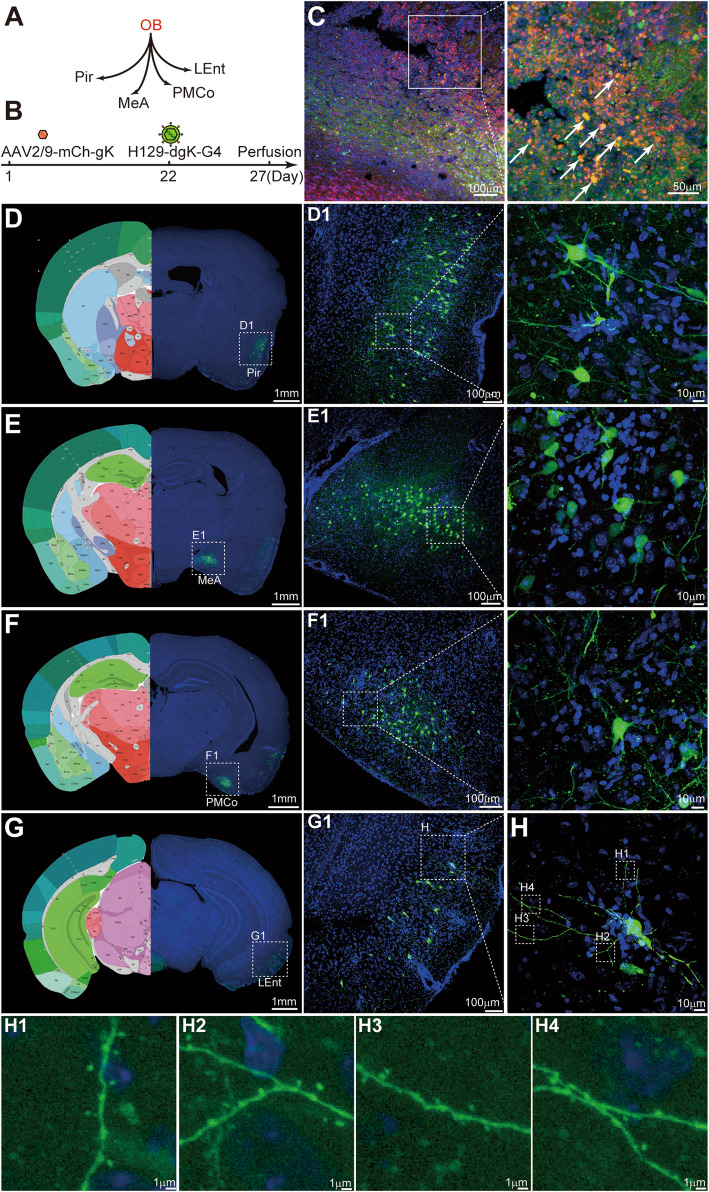
Anterograde monosynaptic tracing of H129-dgK-G4. **A.** Schema of the simplified OB projection pathways. OB; olfactory bulb; Pir, piriform cortex; MeA, medial amygdaloid nucleus, anterior part; PMCo, posteromedial cortical amygdaloid nucleus; LEnt, lateral entorhinal cortex. **B**. The tracing timeline. Helper virus AAV2/9-mCh-gK (1.0 × 10^12^ vg/ml, 150 nl) and H129-dgK-G4 (5.0 × 10^8^ pfu/ml, 150 nl) were sequentially injected into the same region of OB in wildtype C57BL/6 mice at day 1 and day 22 respectively. The brains were collected at day 27 and images were obtained after cryosection. **C–G**. Representative tracing results. The representative images of the injection site of OB (**C**) and the downstream projection target brain regions (**D**-**G**) are shown. The boxed areas are presented in the right panels with higher magnification, and the starter neurons labeled by both GFP and mCherry are indicated with white arrows. Representative images shown are of the same brain from 3 mice. The left hemisphere displays the corresponding sections from Allen Brain Reference Atlases (Image credit: Allen Institute) (**D**–**G**). **H**. A representative postsynaptic neuron well labeled by H129-dgK-G4. Images of a representative GFP-labeled post-synaptic neuron in LEnt are shown, and the magnified images of the dendritic segments are presented (H1-H4)

The features of gK_mut_ pseudotyped tracer H129-dgK-G4 include stronger labeling intensity, higher tracing efficiency, and higher anterograde specificity as described above. The anterograde monosynaptic tracing of H129-dgK-G4 was firstly tested in the olfactory circuit (Fig. 5A). AAV2/9-mCh-gK (1.0 × 10^12^ vg/ml, 150 nl) and H129-dgK-G4 (5.0 × 10^8^ pfu/ml, 150 nl) were sequentially injected into the olfactory bulb (OB) (AP: − 4.28 mm; ML: − 0.50 mm; DV: − 2.50 mm) of wildtype C57BL/6 mice on day 1 and day 22, respectively, and brains were obtained on day 27 (Fig. 5B). Comparing the tracing results along with the time indicated day 27 as the optimized time point to collect the brains, when desirable labeling intensity and efficiency achieved in the downstream brain regions (Additional file: Fig. S3). Thus the brains were collected on day 27 for all the experiments in the present study, unless specified. Neurons labeled with mCherry and GFP (merged as yellow, indicated with the white arrow) were directly observed around the injected site, which were coinfected by both viruses (Fig. 5C). They also represented the potential starter neurons for transmission initiation, from which the gK compensated H129-dgK-G4 transmitted to and labeled the postsynaptic neurons in the downstream brain regions. The GFP-labeled neurons were observed in the representative OB projecting regions, such as piriform cortex (Pir), medial amygdaloid nucleus, anterior part (MeA), posteromedial cortical amygdaloid nucleus (PMCo), and lateral entorhinal cortex (LEnt) (Fig. 5D-G). Notably, All these GFP^+^ neurons were labeled with strong intensity and directly visible without signal amplification by immunostaining. And contributed by the bright labeling intensity and mGFP, the morphology and fine structure details, such as dendritic spines, were also visible in the labeled postsynaptic neurons (Fig. 5H). Injection of AAV2/9-mCh-gK or H129-dgK-G4 alone only labeled neurons around the injection sites (OB) but not the connected regions, indicating neither non-specific transmission nor retrograde labeling occurred under this experimental condition (Additional file: Fig. S4), which guaranteed the specificity of the anterograde monosynaptic tracing of H129-dgK-G4. These data also indicate that H129-dgK-G4, but not the AAV helper, transmits to the postsynaptic neurons from the coinfected started neurons.

To precisely map the neuronal circuits, output information from a specific type of neuron is also required. In mice, the Cre/lox recombination system is the most widely used approach to access specific neuron types. So we further tested the starter-specific anterograde monosynaptic tracing ability of the H129-dgK-G4 system in GAD2-Cre transgenic mice, which specifically express Cre recombinase in neurons with glutamic acid decarboxylase 2 (GAD2). AAV2/9-DIO-mCh-gK was applied as the helper virus to assist the Cre-dependent anterograde monosynaptic tracing of H129-dgK-G4 from a specific neuron type. Controlled by the double floxed inverted orientation (DIO) Cre-On system, AAV2/9-DIO-mCh-gK expresses mCherry and gK only in the presence of Cre recombinase, therefore allowing H129-dgK-G4 monosynaptic transmission only from the Cre expressing starter neurons (Fig. 1C).

**Fig. 6:**
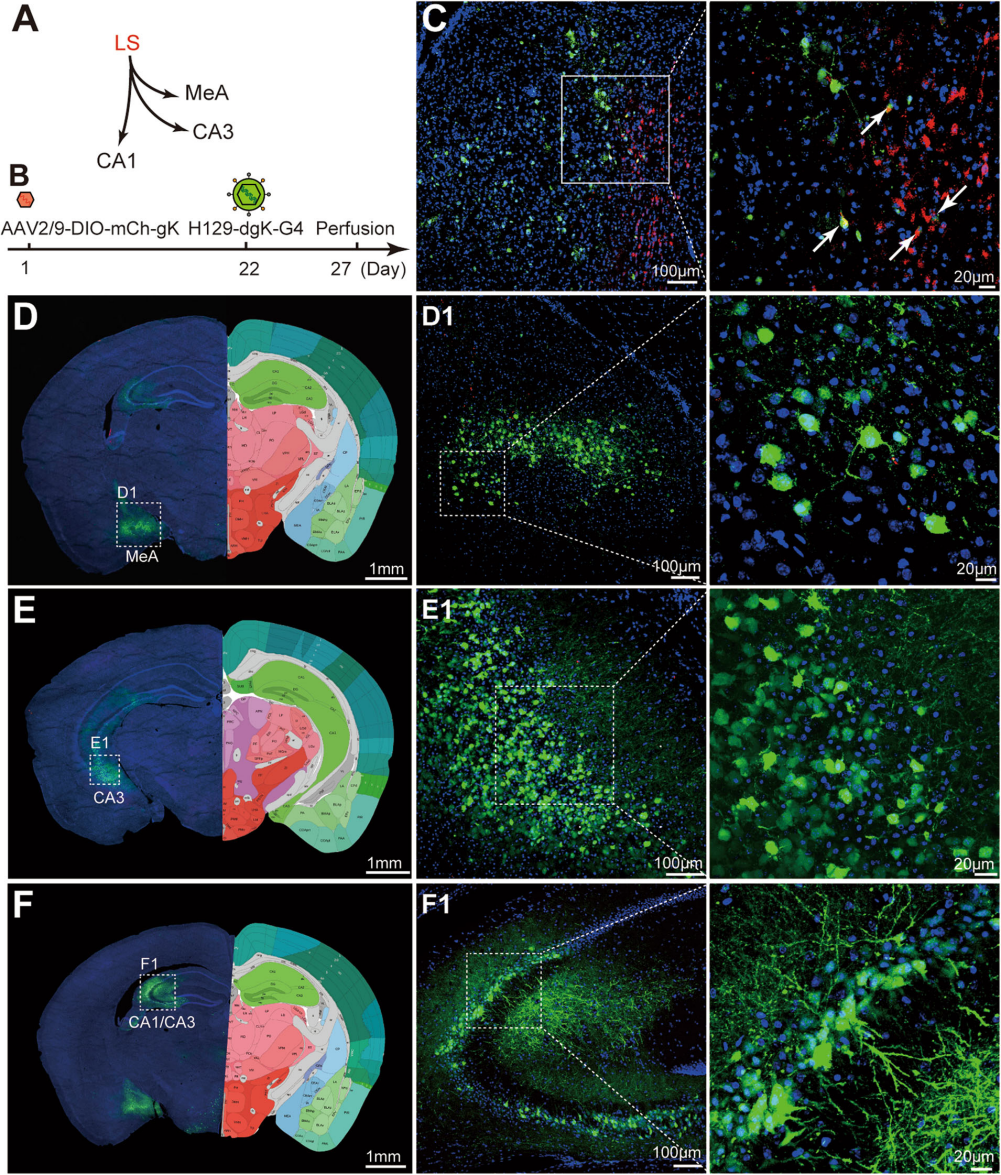
Cre-dependent anterograde monosynaptic tracing of H129-dgK-G4. **A.** Schema of the simplified lateral septal nucleus (LS) projection pathways. LS, lateral septal nucleus; CA1, field CA1 of hippocampus; CA3, field CA3 of hippocampus; MeA, medial amygdaloid nucleus, anterior part. **B**. The tracing timeline. Helper virus AAV2/9-DIO-mCh-gK (1.0 × 10^12^ vg/ml, 100 nl) and H129-dgK-G4 (5.0 × 10^8^ pfu/ml, 100 nl) were sequentially injected into the same region of LS in GAD2-Cre transgenic mice at day 1 and day 22, respectively. The brains were collected at day 27 and images were obtained after cryosection. **C-F**. Representative tracing results. The representative images of the injection site of LS (**C**) and the downstream projection target brain regions (**D**–**F**) are shown. The boxed areas are presented in the right panels with higher magnification, and the starter neurons labeled by both GFP and mCherry are indicated with white arrows. Representative images shown are of the same brain from 3 mice. The right hemisphere displays the corresponding sections from Allen Brain Reference Atlases (Image credit: Allen Institute) (**D**–**F**)

The lateral septal nucleus (LS), containing abundant GAD2 positive neurons, projects to MeA and hippocampus CA1/CA3 (Fig. 6A), and was chosen as the injection site (AP: + 0.74 mm; ML: − 0.30 mm; DV: − 3.58 mm). AAV2/9-DIO-mCh-gK (1.0 × 10^12^ vg/ml, 150 nl) and H129-dgK-G4 (5.0 × 10^8^ pfu/ml, 150 nl) were sequentially injected into the LS of GAD2-Cre mice on day 1 and day 22 respectively. On day 27, the brains were obtained after perfusion and processed for imaging (Fig. 6B). Neurons co-labeled with mCherry and GFP were observed around the injection site, representing the potential starter neurons that were infected by both viruses (Fig. 6C, indicated with the white arrows). Abundant neurons in the downstream brain regions were observed, such as the MeA and the CA3/CA1 of the hippocampus (Fig. 6D-F). Injecting AAV2/9-DIO-mCh-gK or H129-dgK-G4 alone only labeled neurons around the injection sites of LS, but not in the connected regions (Additional file: Fig. S5), which confirmed the specificity of anterograde monosynaptic tracing.

These results demonstrated that the gK_mut_ pseudotyped tracer H129-dgK-G4 is capable of performing specific anterograde monosynaptic tracing with strong labeling intensity and tracing efficiency, both in wildtype mice and in a starter specific manner in Cre-transgenic mice.

### Decreased mPFC-CoA connections in AD mouse brains revealed by H129-dgK-G4 tracing

Neural network abnormalities are involved in many brain diseases. Alzheimer’s disease and Parkinson’s disease usually display neuronal damage and are accompanied by neural circuit changes, and autism is usually associated with neural network abnormalities [32]. So dissecting the differences of neuronal connections between the diseased and healthy individuals is important for understanding the mechanisms of these diseases. The anterograde polysynaptic tracer H129-G4 was previously used to reveal the impaired connectivity from the primary motor cortex (M1) to the subthalamic nucleus (STN) in unilateral 6-hydroxydopamine (6-OHDA)-lesioned parkinsonian rats [33]. However, since H129-G4 is a polysynaptic tracer, the comparison had to be performed by limiting the transmission time to avoid potential detoured tracing, which is not capable of precisely controlling the transmission order [26, 34, 35]. H129-dgK-G4 offers a better tool to achieve a more accurate comparison by its monosynaptic tracing specificity.

**Fig. 7:**
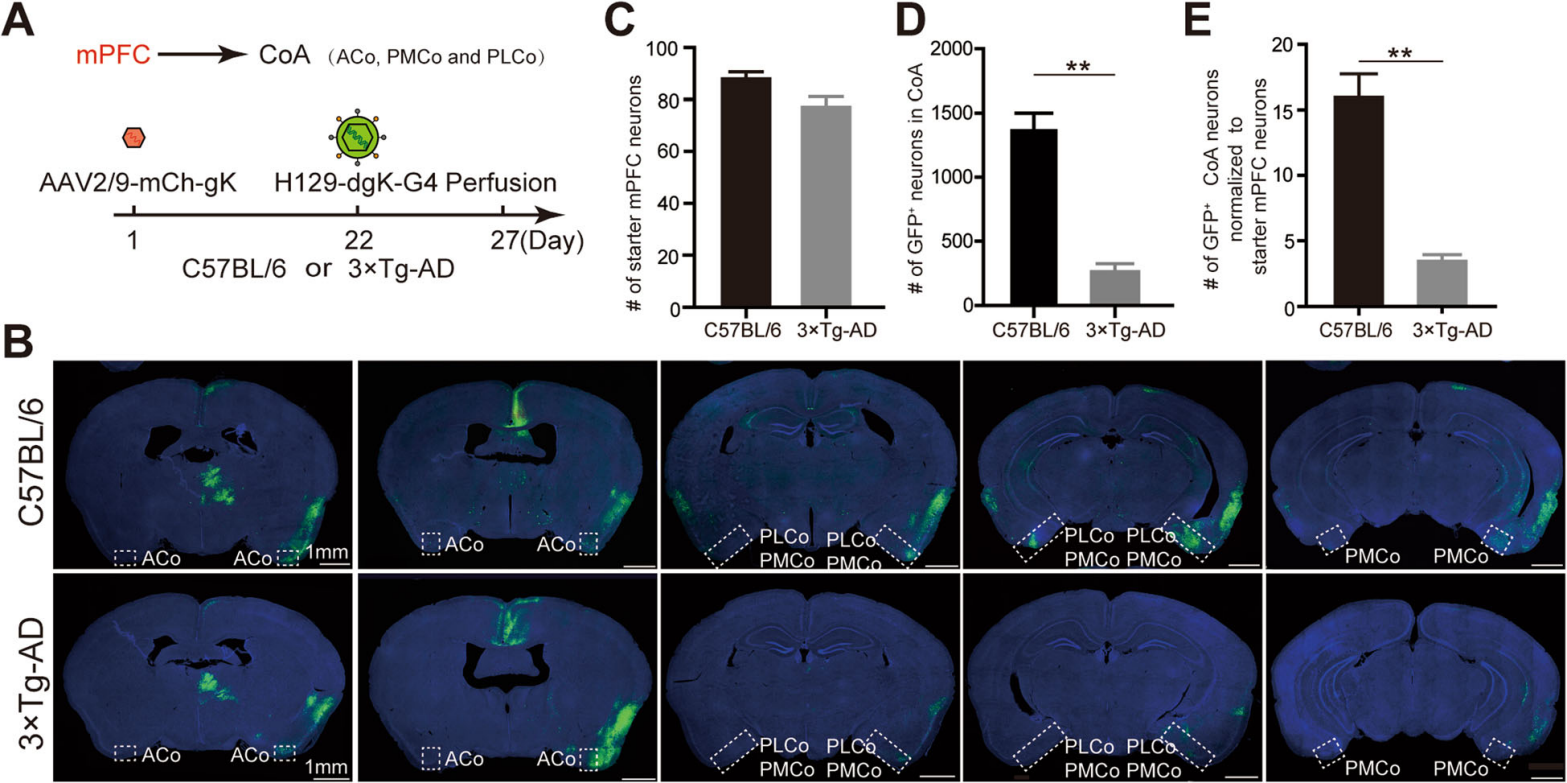
Quantitative comparison of mPFC-CoA connections between Alzheimer’s disease and control mice by tracing with H129-dgK-G4. **A.** The tracing timeline. Helper virus AAV2/9-mCh-gK (1.0 × 10^12^ vg/ml, 150 nl) and H129-dgK-G4 (5.0 × 10^8^ pfu/ml, 150 nl) were sequentially injected into the medial prefrontal cortex (mPFC) of wildtype C57BL/6 mice and 3 × Tg-AD mice at day 1 and day 22, and the brains were perfused and collected at day 27. CoA, cortical amygdaloid nucleus. **B.** Representative tracing results. Representative images of the labeled neurons in CoA (indicated with the dashed boxes) of the wildtype C57BL/6 and 3 × Tg-AD mice are shown. ACo, anterior cortical amygdaloid nucleus; PMCo, posteromedial cortical amygdaloid nucleus; PLCo, posterolateral cortical amygdaloid nucleus. **C–E.** Quantitative analysis. The numbers of mCherry/GFP double-labeled starter neurons in mPFC (**C**) and the GFP-labeled postsynaptic neurons in CoA (including Aco, PMCo, and PLCo in both hemispheres) (**D**) were counted in three mice for each group and shown. The statistical significance was analyzed by LME. **, *p* < 0.01. The average ratio of GFP^+^ CoA neurons to mPFC starter neurons was calculated and shown, and the statistical significance was analyzed by Student’s t-test. **, *p* < 0.01 (**E**)

3 × Tg-AD mouse is a broadly-used Alzheimer’s disease model, which contains multiple mutations (APP Swedish, MAPT P301L, and PSEN1 M146V) associated with familial Alzheimer’s disease [36]. At the age of 3-month-old, the 3 × Tg-AD mouse showed a significantly decrease level of synaptophysin in the cortex [37]. The projection from the medial prefrontal cortex (mPFC) to the cortical amygdaloid nucleus (CoA) has been reported in rat and macaque monkeys [38, 39]. Our preliminary data in the mouse brain also showed strong projection from mPFC to CoA (data not shown). To compare the synaptic connectivity between the cortex and other brain regions, we sequentially injected the AAV2/9-mCh-gK (1.0 × 10^12^ vg/ml, 150 nl) and H129-dgK-G4 (5.0 × 10^8^ pfu/ml, 150 nl) into mPFC (AP: + 1.78 mm; ML: − 0.16 mm; DV: − 3.00 mm) of 3-month-old 3 × Tg-AD or the control wildtype C57BL/6 mice at the same age (Fig. 7A). The labeled cells were quantitated in CoA, which is one direct innervating region of mPFC. The representative images clearly showed fewer CoA neurons were labeled by H129-dgK-G4 in 3 × Tg-AD than those in the wildtype mice (Fig. 7B).

Although the starter neurons coinfected by AAV-mCh-gK and H129-dgK-G4 are damaged by viral tracer replication, they still maintain intact cell morphology and can be observed by colabeled fluorescent proteins at day 5 post the tracer injection. This makes it possible to identify the starter neuron and analyze the connectivity by comparing the tracer-labeled postsynaptic neurons to the colabeled starter neurons. We counted both numbers of mCherry/GFP double-labeled neurons in the mPFC as starter neurons, and the GFP-labeled postsynaptic neurons in CoA of both hemispheres, including the anterior cortical amygdaloid nucleus (ACo), posteromedial cortical amygdaloid nucleus (PMCo), and posterolateral cortical amygdaloid nucleus (PLCo). AAV helper and H129-dgK-G4 labeled similar amount of starter neurons in mPFC of wildtype and 3 × Tg-AD mice (86 ± 2 vs 76 ± 4) (Fig. 7C). Whereas, dramatic differences were observed in the amount of the postsynaptically labeled CoA neurons. In wildtype mice, an average of 1363 ± 140 GFP^+^ neurons were observed in CoA of each mouse brain, whereas there are only 259 ± 53 GFP^+^ neurons in CoA of each 3 × Tg-AD mouse brain (Fig. 7D). When normalizing the GFP^+^ CoA neurons to the mPFC starter neuron in each mouse, 3 × Tg-AD mouse clearly showed a significantly decreased mPFC-CoA connection compared to the wildtype control (3.4 ± 0.6 vs 15.9 ± 1.8) (Fig. 7E). On the contrary, neither the GFP^+^ neurons amount in the thalamus (76 ± 9 vs 81 ± 8) nor the ratio of GFP^+^ thalamic neuron to mPFC starter neuron (0.9 ± 0.1 vs 1.0 ± 0.1) showed significant differences between wildtype and 3 × Tg-AD mice (Additional file: Fig. S6). These results indicate that the mPFC-CoA connectivity in this Alzheimer’s disease model at the age of 3-month old was decreased by 81% compared to the wildtype control, whereas the mPFC-thalamus connection was not altered. This is also consistent with the reduced cortex synaptophysin of the 3 × Tg-AD mouse reported previously [37]. Therefore, H129-dgK-G4 could potentially be a powerful tool to quantify direct neuronal connectivity.

## Discussion

Prior to H129-dgK-G4, the applicable anterograde monosynaptic tracers included AAV1 and the TK deficient H129 tracers (H129-dTK-tdT and H129-dTK-2 T) [5, 10, 13]. Successful application of AAV1 anterograde tracing requires a high virus titer (≥1.0 × 10^13^ vg/ml) and signal amplification with a reporter system to overcome the low transneuronal transmission efficiency. Another always ignored but inevitable drawback of AAV1 is its potential invasion through the axon terminal, which resulted in retrograde labeling in the upstream brain region, especially under such a high administration titer [5, 40, 41]. In addition, the incapability to replicate and the viral transmission mechanism doesn’t allow AAV1 for starter neuron-specific tracing [40].

Our previously developed anterograde monosynaptic tracers H129-dTK-tdT and H129-dTK-T2 are derived from H129 and based on TK deficiency, which leads to certain intrinsic drawbacks such as low labeling intensity, low labeling efficiency, and potential retrograde labeling [10, 12, 13]. H129-dTK-G4 used in the present study had similar labeling intensity to H129-dTK-T2. All these data indicate that the labeling intensity has hit the ceiling by the TK deficiency strategy, and a different strategy is required to improve the H129-based anterograde monosynaptic tracers. By targeting gK, we created the novel anterograde monosynaptic tracer H129-dgK-G4, which overcomes these limitations at least partially.

Targeting gK increases the labeling intensity and tracing efficiency. The glycoprotein gK is a structural protein, expresses at the late stage of viral replication, resides on the envelope at the very last step of viral assembly. Therefore, its deficiency doesn’t influence viral genome replication, viral protein synthesis, and primary viral assembly, but impairs virus egress and transmission. Compared to the TK-deficient H129 monosynaptic tracers, the level of intrinsic fluorescent protein expression of H129-dgK-G4 is dramatically increased in the labeled postsynaptic neurons. The two copies of the tandem mGFP-GFP expression cassette further boost the fluorescent protein expression level, resulting in stronger labeling intensity. Moreover, mGFP, which binds to the member of the infected cells, greatly improves the H129-dgK-G4 labeling effect by well visualizing the cell membrane and displays the detailed structure. Contributed by the stronger labeling intensity, H129-dgK-G4 also labels and visualizes more postsynaptic neurons in the downstream brain regions, and thus displays higher anterograde monosynaptic tracing efficiency.

Target gK by pseudotyping with gK_mut_ can also help to improve the specificity of anterograde tracing. gK is related to viral egress, viral particles axonal transport, virus-induced cell fusion, as well as infection via axon invasion [20, 21, 23, 42]. It was reported that recombinant HSV-1 coated with the gK protein from strain KOS dramatically decreased infection efficiency through the axon invasion than the virus coated with gK from strain McKrae [23]. The interaction between gK and gB might affect gB mediating fusion of the viral envelope with cellular membranes during virus entry [20, 43, 44]. Although the exact mechanism remains to be revealed, the amino acid difference of gK between different HSV-1 strains is associated with viral axon invasion. Therefore, we accordingly mutated the gK from the H129 strain, and generated the Vero-gK_mut_ cells stably expressing the gK_mut_ (A40V, C82S, M223I, L224V, V309M) for preparation of viral tracer pseudotyped with gK_mut_. The retrograde labeling ratio of H129-dgK-G4 coated with gK_mut_ and gK_wt_ was compared. The results confirmed that gK_mut_ pseudotyping significantly reduced the retrograde labeling by 77% in vitro and in vivo. Thus, H129-dgK-G4 was propagated in Vero-gK_mut_ to produce the gK_mut_ pseudotyped viral tracer with lower retrograde labeling. While, AAV helper expressing gK_wt_ was applied to achieve better viral yield in the coinfected starter neurons, which will further facilitate the tracer transneuronal transmission efficiency. Notably, although gK_mut_ pseudotyping greatly reduced the retrograde labeling, it failed to completely eliminate the terminal invasion. Mutations on other gK amino acids might achieve further improvement, but a strategy similar to the EnvA/TVA system of the rabies virus tracer represents a better strategy to solve the retrograde labeling issue [45]. Targeting gK and pseudotyping with gK_mut_ can clearly reduce the retrograde labeling caused by axon terminal invasion, thus enhance the anterograde specificity of H129-dgK-G4 monosynaptic tracing.

The low abundance of gK benefits the function restoration and production of tracer H129-dgK-G4. If the deficient target is an abundant viral essential protein, it requires a higher compensatory level to restore the function. An insufficient protein level expressed by AAV will limit the viral yield and transmission efficiency. Compared to other viral structural proteins, gK possesses a relatively low abundance in virion and low expression level in infected cells [17]. Low abundant gK can be easily compensated by the AAV helper. The efficient propagation of H129-dgK-G4 in gK expressing cell lines yields desired titer of H129-dgK-G4 (5 × 10^8^ pfu/ml). H129-dgK-G4 along with the AAV helper can label downstream neurons in vitro and efficiently trace the projection neurons in downstream brain regions in vivo. All these data support that gK expressing cells and AAV helper can provide sufficient gK compensation, which avoids hurdles from gK deficiency and will benefit the tracer production as a tracer resource.

Among currently monosynaptic anterograde tracers which have been reported and applied, H129-dgK-G4 shows the strongest labeling intensity, the best tracing efficiency, and the lowest retrograde labeling ratio. The abundant and fast expression of the fluorescent protein also shortens the experiment duration. For the TK deficient H129 monosynaptic anterograde tracers, 7–10 days are required to visualize the postsynaptic neurons in downstream brain regions after the injection H129-dTK-tdT or H120-dTK-T2 [10, 13]. But contributed by the strong labeling intensity, H129-dgK-G4 is capable of well labeling the postsynaptic neurons in 5 days post its injection. At this earlier time point, the co-infected starter neurons at the injection site are less severely damaged, and better cell morphology and conditions can be observed.

Abnormalities of the neuronal networks are often observed in Alzheimer’s disease, Parkinson’s disease, and Autism [32]. Viral circuit tracers have contributed to better displaying the abnormalities of the neuronal networks under the diseases condition. H129-G4 was applied to compare connectivity between parkinsonian and healthy rats. But since H129-G4 is a polysynaptic tracer that may spread through multiple orders, so controlling the tracing time is the only method to achieve a similar effect of monosynaptic tracing, which obviously is less precise [26, 34, 35]. By using the TK deficient anterograde monosynaptic tracers, postsynaptic neurons can usually be visualized at 7–10 days post tracer injection [10, 13]. While at this time point, replication of the viral tracer already causes severe damage to the starter neurons, which are colabeled by GFP (from helper AAV) and tdTomato (from H129-dTK tracers), and lost the normal cell morphology [10]. The observing time gap for starter neurons (3–5 days post tracer injection) and postsynaptic neurons (7–10 days post tracer injection) makes it difficult to observe the starter and postsynaptic neurons at the same time. So H129-dTK tracers are not feasible for quantitative connectivity analysis. The novel tracer H129-dgK-G4 shortens the tracing time to 5 days, when the postsynaptic neurons have been brightly labeled, and the starter neurons still maintain relatively intact morphology and the colabeling by tracer and helper are visible. Thus, H129-dgK-G4 makes it possible for quantitative connectivity analysis by normalizing the number of H129-dgK-G4 labeled postsynaptic neurons to the number of AAV-mCh-gK and H129-dgK-G4 colabeled starter neurons. In this study, we applied H129-dgK-G4 in 3 × Tg-AD mice, and revealed 81% decreased mPFC-CoA connection in the mice with Alzheimer’s disease. This showed that H129-dgK-G4 provides the first anterograde tracing tool which is capable of performing qualitative analysis and comparison for the direct output connections precisely.

Although H129-dgK-G4 has displayed multiple improved tracing properties, it remains one intrinsic drawback shared by all the H129-derived tracers, which is the relatively high toxicity to the infected cells [12]. In the starter neurons, AAV helper complementarily expressing gK assists the replication of H129-dgK-G4, which causes severe damage to the neurons. And in the postsynaptic neurons, H129-dgK-G4 alone also hijacks the cellular machinery for viral genome replication and viral proteins synthesis, which dramatically affects normal cellular physiological function and cell viability. Therefore, H129-dgK-G4 tracer is powerful for anatomic mapping, but not suitable for functional tracing analysis, such as Ca^2+^ imaging or optogenetic assay. Shortening the time between H129-dgK-G4 injection and brain collection might contribute to slightly reducing the cell damage, but it will also compromise labeling efficiency and intensity. The mechanism of H129-induced cytotoxicity needs to be investigated, and further systematic work on H129 attenuation is also urgently required for developing improved H129-derived tracers compatible with functional assays.

## Conclusions

By targeting gK, we have generated H129-dgK-G4, a novel anterograde monosynaptic tracer with multiple advantages. It has strong labeling intensity, high tracing efficiency, and reduced retrograde labeling, which make H129-dgK-G4 an improved tool for anterogradely tracing the direct output network. We believe H129-dgK-G4 represents one of the best monosynaptic anterograde tracers so far, and will contribute to deciphering the neuronal connectome and disease mechanism.

## Supplementary Information

**Additional File 1.**

## Data Availability

The datasets used and/or analyzed during the current study are available from the corresponding author on reasonable request.

## Abbreviations

HSV-1: Herpes simplex virus 1
H129: Herpes simplex virus 1 (HSV-1) strain H129
*gK*: glycoprotein K gene
gK_wt_: wildtype glycoprotein K of H129
gK_mut_: mutant glycoprotein K (A40V, C82S, M223I, L224V, V309M)
Vero: Vero-E6 cell
Vero-gK_wt_: Vero cell line stably expressing wildtype gK
Vero-gK_mut_: Vero cell line stably expressing the mutant gK
tdT: tdTomato
mCh: mCherry
*TK*: thymidine kinase gene
DMEM: Dulbecco’s modified Eagle medium
HBSS: Hanks Balanced Salt Solution
mGFP: membrane-bound EGFP
*Amp*^*R*^: Ampicillin resistant gene
*Zeo*^*R*^: Zeocin resistant gene
Cre: Cre recombinase
DIO: double floxed inverted orientation
ML: Mediolateral
AP: Anteroposterior
DV: Dorsoventral
PFA: Paraformaldehyde
GAD2: glutamic acid decarboxylase 2
AU: arbitrary unit
M1: primary motor cortex
Au: auditory cortex
DG: dentate gyrus
V1: visual cortex
OB: olfactory bulb
Ect: ectorhinal cortex
LD: laterodorsal thalamic nucleus
SC: superior colliculus
LGd: dorsal lateral geniculate nucleus
LS: lateral septal nucleus
MeA: medial amygdaloid nucleus, anterior part
6-OHDA: unilateral 6-hydroxydopamine
CoA: cortical amygdaloid nucleus
LGN: lateral geniculate nucleus
Pir: piriform cortex
mPFC: medial prefrontal cortex
dpi: day post infection
RABV: Rabies virus

## References

[1] Xu X, Holmes TC, Luo MH, Beier KT, Horwitz GD, Zhao F, Zeng W, Hui M, Semler BL, Sandri-Goldin RM (2020). Viral vectors for neural circuit mapping and recent advances in trans-synaptic anterograde tracers. Neuron.

[2] Li J, Liu T, Dong Y, Kondoh K, Lu Z (2019). Trans-synaptic neural circuit-tracing with neurotropic viruses. Neurosci Bull.

[3] Nassi JJ, Cepko CL, Born RT, Beier KT (2015). Neuroanatomy goes viral!. Front Neuroanat.

[4] Callaway EM, Luo L (2015). Monosynaptic circuit tracing with glycoprotein-deleted rabies viruses. J Neurosci.

[5] Zingg B, Chou XL, Zhang ZG, Mesik L, Liang F, Tao HW, Zhang LI (2017). AAV-mediated anterograde Transsynaptic tagging: mapping Corticocollicular input-defined neural pathways for defense behaviors. Neuron.

[6] McGovern AE, Davis-Poynter N, Rakoczy J, Phipps S, Simmons DG, Mazzone SB (2012). Anterograde neuronal circuit tracing using a genetically modified herpes simplex virus expressing EGFP. J Neurosci Methods.

[7] Lo L, Anderson DJ (2011). A Cre-dependent, anterograde transsynaptic viral tracer for mapping output pathways of genetically marked neurons. Neuron.

[8] Everett RD (2014). HSV-1 biology and life cycle. Methods Mol Biol.

[9] Mcgovern AE, Davis-Poynter N, Farrell MJ, Mazzone SBJN. Transneuronal tracing of airways-related sensory circuitry using herpes simplex virus 1, strain H129. 2012, 207:148–166. 10.1016/j.neuroscience.2012.01.029 22306285

[10] Zeng WB, Jiang HF, Gang YD, Song YG, Shen ZZ, Yang H, Dong X, Tian YL, Ni RJ, Liu Y, Tang N, Li X, Jiang X, Gao D, Androulakis M, He XB, Xia HM, Ming YZ, Lu Y, Zhou JN, Zhang C, Xia XS, Shu Y, Zeng SQ, Xu F, Zhao F, Luo MH (2017). Anterograde monosynaptic transneuronal tracers derived from herpes simplex virus 1 strain H129. Mol Neurodegener.

[11] Su P, Ying M, Han Z, Xia J, Jin S, Li Y, Wang H, Xu F (2020). High-brightness anterograde transneuronal HSV1 H129 tracer modified using a Trojan horse-like strategy. Mol Brain.

[12] Li D, Yang H, Xiong F, Xu X, Zeng WB, Zhao F, et al. Anterograde neuronal circuit tracers derived from herpes simplex virus 1: development, application, and perspectives. Int J Mol Sci. 2020;21(16). 10.3390/ijms21165937. 10.3390/ijms21165937 PMC7460661 32824837

[13] Yang H, Xiong F, Song YG, Jiang HF, Qin HB, Zhou J, Lu S, Grieco SF, Xu X, Zeng WB, Zhao F, Luo MH (2020). HSV-1 H129-derived anterograde neural circuit tracers: improvements, production, and applications. Neurosci Bull.

[14] Munch-Petersen B (2010). Enzymatic regulation of cytosolic thymidine kinase 1 and mitochondrial thymidine kinase 2: a mini review. Nucleosides Nucleotides Nucleic Acids.

[15] Wojaczynski GJ, Engel EA, Steren KE, Enquist LW, Patrick Card J (2015). The neuroinvasive profiles of H129 (herpes simplex virus type 1) recombinants with putative anterograde-only transneuronal spread properties. Brain Struct Funct.

[16] Su P, Wang H, Xia J, Zhong X, Hu L, Li Y, Li Y, Ying M, Xu F (2019). Evaluation of retrograde labeling profiles of HSV1 H129 anterograde tracer. J Chem Neuroanat.

[17] Birzer A, Kraner ME, Heilingloh CS, Muhl-Zurbes P, Hofmann J, Steinkasserer A, Popella L (2020). Mass spectrometric characterization of HSV-1 L-particles from human dendritic cells and BHK21 cells and analysis of their functional role. Front Microbiol.

[18] Melancon JM, Luna RE, Foster TP, Kousoulas KG (2005). Herpes simplex virus type 1 gK is required for gB-mediated virus-induced cell fusion, while neither gB and gK nor gB and UL20p function redundantly in virion de-envelopment. J Virol.

[19] Jayachandra S, Baghian A, Kousoulas KG (1997). Herpes simplex virus type 1 glycoprotein K is not essential for infectious virus production in actively replicating cells but is required for efficient envelopment and translocation of infectious virions from the cytoplasm to the extracellular space. J Virol.

[20] Jambunathan N, Chowdhury S, Subramanian R, Chouljenko VN, Walker JD, Kousoulas KG (2011). Site-specific proteolytic cleavage of the amino terminus of herpes simplex virus glycoprotein K on virion particles inhibits virus entry. J Virol.

[21] Foster TP, Rybachuk GV, Kousoulas KG (2001). Glycoprotein K specified by herpes simplex virus type 1 is expressed on virions as a Golgi complex-dependent glycosylated species and functions in virion entry. J Virol.

[22] Foster TP, Kousoulas KG (1999). Genetic analysis of the role of herpes simplex virus type 1 glycoprotein K in infectious virus production and egress. J Virol.

[23] David AT, Saied A, Charles A, Subramanian R, Chouljenko VN, Kousoulas KG: A herpes simplex virus 1 (McKrae) mutant lacking the glycoprotein K gene is unable to infect via neuronal axons and egress from neuronal cell bodies. mBio 2012, 3:e00144–00112. 10.1128/mBio.00144-12 PMC3413403 22829677

[24] Yang B, Liu XJ, Yao Y, Jiang X, Wang XZ, Yang H, et al. WDR5 facilitates human cytomegalovirus replication by promoting capsid nuclear egress. J Virol. 2018;92(9). 10.1128/JVI.00207-18. 10.1128/JVI.00207-18 PMC5899187 29437978

[25] Yang Y, Chen J, Guo Z, Deng S, Du X, Zhu S, Ye C, Shi YS, Liu JJ (2018). Endophilin A1 promotes actin polymerization in dendritic spines required for synaptic potentiation. Front Mol Neurosci.

[26] Dong X, Zhou J, Qin HB, Xin B, Huang ZL, Li YY, et al. Anterograde viral tracer herpes simplex virus 1 strain H129 transports primarily as capsids in cortical neuron axons. J Virol. 2020;94(8). 10.1128/JVI.01957-19. 10.1128/JVI.01957-19 PMC7108832 31969440

[27] Paxinos G, Franklin KBJ (2004). The mouse brain in stereotaxic coordinates.

[28] Liu YJ, Spangenberg EE, Tang B, Holmes TC, Green KN, Xu X (2021). Microglia elimination increases neural circuit connectivity and activity in adult mouse cortex. J Neurosci.

[29] Grieco SF, Qiao X, Zheng X, Liu Y, Chen L, Zhang H, Yu Z, Gavornik JP, Lai C, Gandhi SP, Holmes TC, Xu X (2020). Subanesthetic ketamine reactivates adult cortical plasticity to restore vision from amblyopia. Curr Biol.

[30] Jiang HF, Wang W, Jiang X, Zeng WB, Shen ZZ, Song YG, et al. ORF7 of varicella-zoster virus is required for viral cytoplasmic envelopment in differentiated neuronal cells. J Virol. 2017;91(12). 10.1128/JVI.00127-17. 10.1128/JVI.00127-17 PMC5446663 28356523

[31] Katz LC, Crowley JC (2002). Development of cortical circuits: lessons from ocular dominance columns. Nat Rev Neurosci.

[32] Insel TR (2010). Rethinking schizophrenia. Nature.

[33] Wang YY, Wang Y, Jiang HF, Liu JH, Jia J, Wang K, Zhao F, Luo MH, Luo MM, Wang XM (2018). Impaired glutamatergic projection from the motor cortex to the subthalamic nucleus in 6-hydroxydopamine-lesioned hemi-parkinsonian rats. Exp Neurol.

[34] Deng K, Yang L, Xie J, Tang H, Wu GS, Luo HR. Whole-brain mapping of projection from mouse lateral septal nucleus. Biol Open. 2019;8. 10.1242/bio.043554. 10.1242/bio.043554 PMC6679409 31208998

[35] Tang H, Wu GS, Xie J, He X, Deng K, Wang H, Xu F, Luo HR (2016). Brain-wide map of projections from mice ventral subiculum. Neurosci Lett.

[36] Oddo S, Caccamo A, Shepherd JD, Murphy MP, Golde TE, Kayed R, Metherate R, Mattson MP, Akbari Y, LaFerla FM (2003). Triple-transgenic model of Alzheimer's disease with plaques and tangles: intracellular Abeta and synaptic dysfunction. Neuron.

[37] Baazaoui N, Iqbal K (2017). Prevention of dendritic and synaptic deficits and cognitive impairment with a neurotrophic compound. Alzheimers Res Ther.

[38] Stefanacci L, Amaral DG (2002). Some observations on cortical inputs to the macaque monkey amygdala: an anterograde tracing study. J Comp Neurol.

[39] McDonald AJ, Mascagni F, Guo L (1996). Projections of the medial and lateral prefrontal cortices to the amygdala: a Phaseolus vulgaris leucoagglutinin study in the rat. Neuroscience.

[40] Zingg B, Peng B, Huang J, Tao HW, Zhang LI (2020). Synaptic specificity and application of anterograde Transsynaptic AAV for probing neural circuitry. J Neurosci.

[41] Rothermel M, Brunert D, Zabawa C, Diaz-Quesada M, Wachowiak M (2013). Transgene expression in target-defined neuron populations mediated by retrograde infection with adeno-associated viral vectors. J Neurosci.

[42] Hutchinson L, Roop-Beauchamp C, Johnson DC (1995). Herpes simplex virus glycoprotein K is known to influence fusion of infected cells, yet is not on the cell surface. J Virol.

[43] Chouljenko VN, Iyer AV, Chowdhury S, Kim J, Kousoulas KG (2010). The herpes simplex virus type 1 UL20 protein and the amino terminus of glycoprotein K (gK) physically interact with gB. J Virol.

[44] Chouljenko VN, Iyer AV, Chowdhury S, Chouljenko DV, Kousoulas KG (2009). The amino terminus of herpes simplex virus type 1 glycoprotein K (gK) modulates gB-mediated virus-induced cell fusion and virion egress. J Virol.

[45] Osakada F, Callaway EM (2013). Design and generation of recombinant rabies virus vectors. Nat Protoc.

