# Supplementary material for: A novel H129-based anterograde monosynaptic tracer exhibits features of strong labeling intensity, high tracing efficiency, and reduced retrograde labeling
Source: Mol Neurodegener. 2022 Jan 10;17:6. doi: 10.1186/s13024-021-00508-6 (PMC8744342; doi:10.1186/s13024-021-00508-6)
Supplement: Supplementary file 1 — Additional File 1. [file 13024_2021_508_MOESM1_ESM.docx]

Additional file

**Figure S1**

**
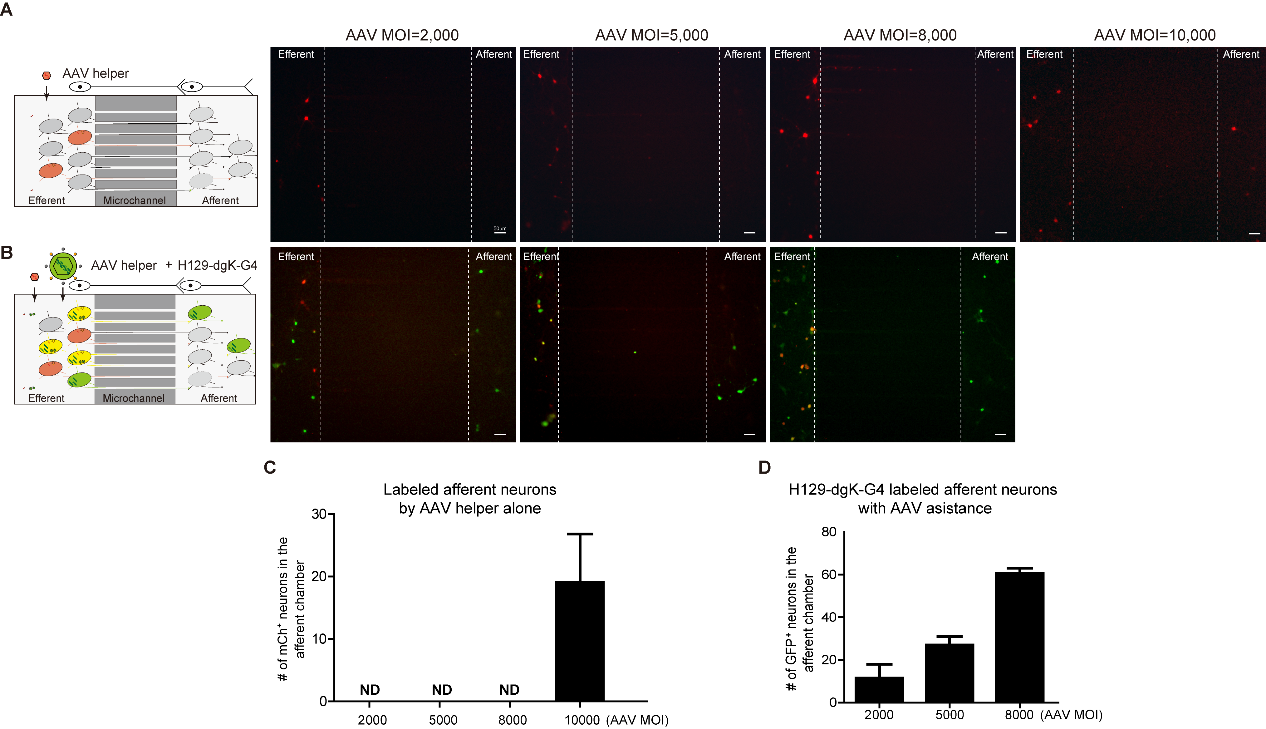
**

**Figure S1.** *In vitro* anterograde monosynaptic labeling by H129-dgK-G4 and the AAV helper.

1×10^6^ fetal mouse cortical neurons were sequentially plated in both chambers of the microfluidic plates on day 1 and day 5, and cultured for additional 14 days to allow synapse formation in the afferent chamber, then add helper AAV with different dosages. The helper AAV, AAV2/9-mCh-gK, were inoculated to the efferent side with MOI of 2000, 5000, 8000, or 10000, respectively (equivalent to the AAV dosage of 2×10^9^ vg, 5×10^9^ vg, 8×10^9^ vg, and 1×10^10^ vg) (A). 7 days later, 1×10^6^ pfu of the tracers, H129-dgK-G4, were inoculated to the efferent chamber (equivalent to MOI of 1) (B). The labeled neurons in the efferent and afferent chamber were examined at 7 days post AAV infection (A) or 2 days post H129-dgK-G4 infection (B). The dotted lines indicate the borders between chambers and the microchannels. The number of mCherry- (C) or GFP-labeled neurons (D) was counted in the afferent chamber, and the average of 5 independent experiments is shown.

**Figure S2**

**
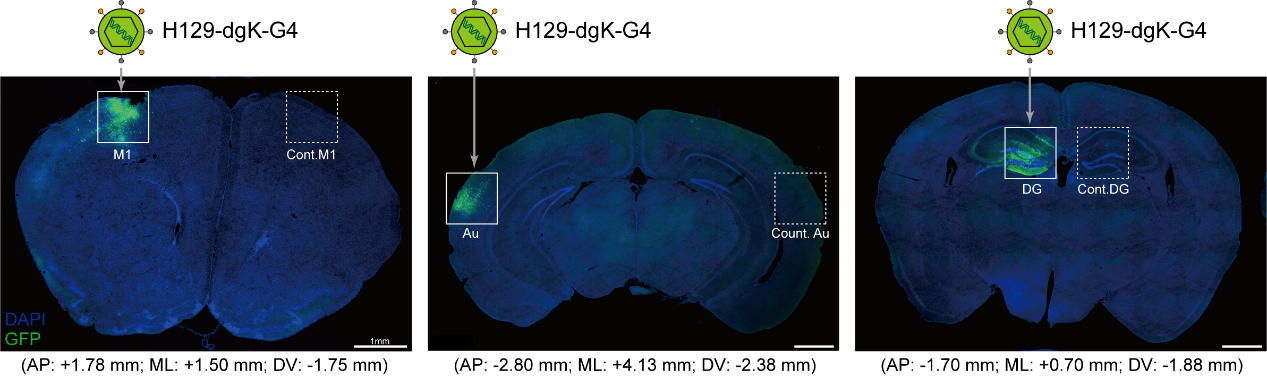
**

**Figure S2.** *In vivo* transneuronal labeling by H129-dgK-G4 alone.

H129-dgK-G4 (5×10^8^ pfu/ml, 100 nl) was injected into the indicated brain regions (indicated by solid-line box) of wildtype C57BL/6 mice. At 5 dpi, the brain samples were collected after perfusion, and the downstream brain regions were examined for GFP labeling. Shown are the representative images of the representative downstream brain regions (indicated by dotted-line box) from 3 mice are shown. M1, primary motor cortex; Cont. M1, contralateral M1; Au, auditory cortex; Cont. Au, contralateral Au; DG, dentate gyrus; Cont. DG, contralateral DG.

**Figure S3**

**
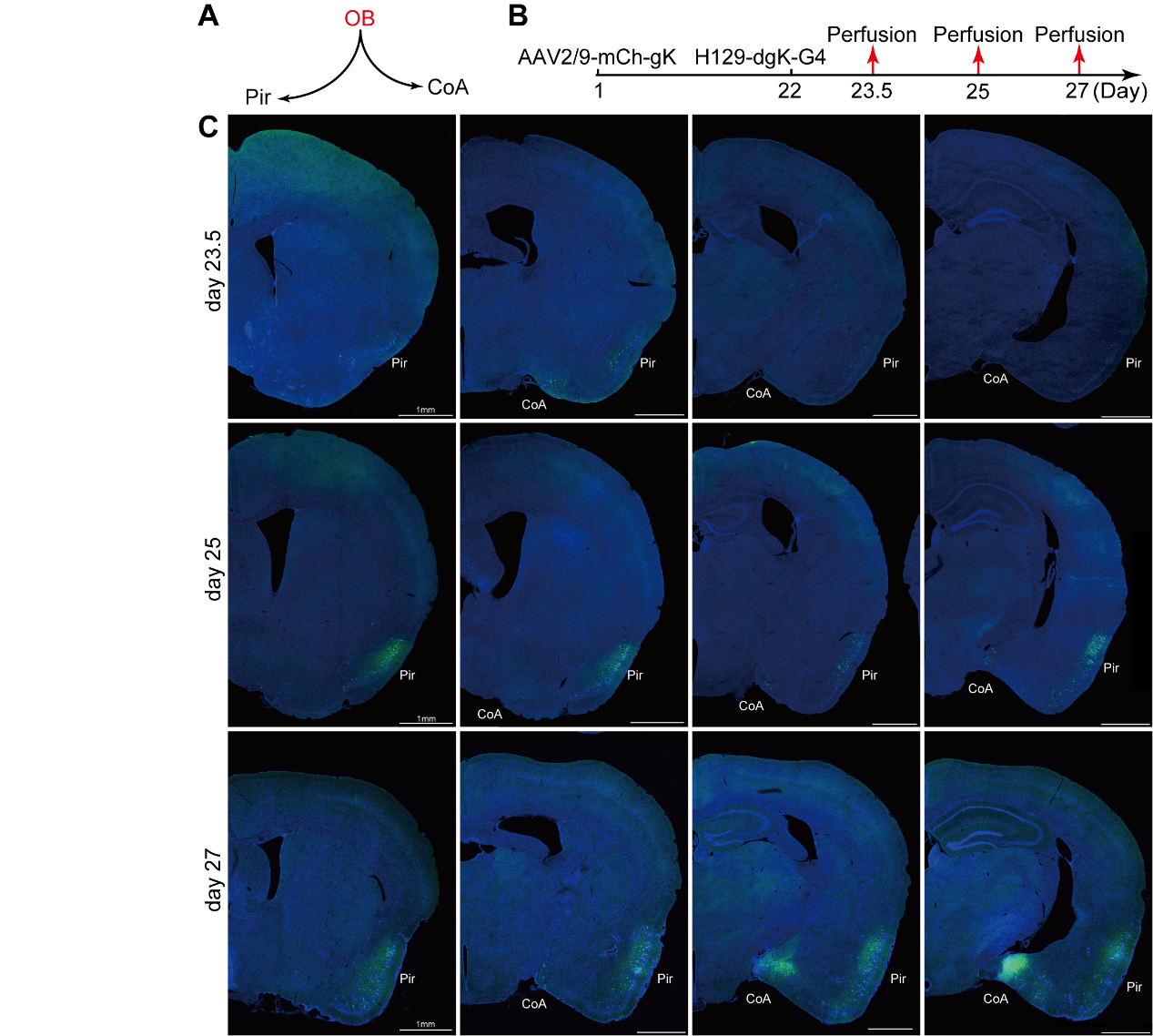
**

**Figure S3.** The timing of H129-dgK-G4 anterograde monosynaptic tracing.

The optimized observation timing of H129-dgK-G4 anterograde monosynaptic tracing was evaluated in olfactory circuits. Schema of the simplified olfactory bulb (OB) projection pathways is shown in A. Helper virus AAV2/9-mCh-gK (1.0×10^12^ vg/ml, 150 nl) and H129-dgK-G4 (5.0×10^8^ pfu/ml, 150 nl) were sequentially injected into the same region of OB (AP: +4.28 mm; ML: +0.50 mm; DV: -2.50 mm) in wildtype C57BL/6 mice at day 1 and day 22, respectively. The brains were collected at day 23.5, day 25, and day 27 (1.5, 3, 5 days post injection of H129-dgK-G4) after perfusion, and images were obtained after cryosection (B). The representative images of OB projecting regions (Pir, CoA) from 3 mice of each group are shown (C). OB, olfactory bulb; Pir, piriform cortex; CoA, cortical amygdaloid nucleus.

**Figure S4**

**
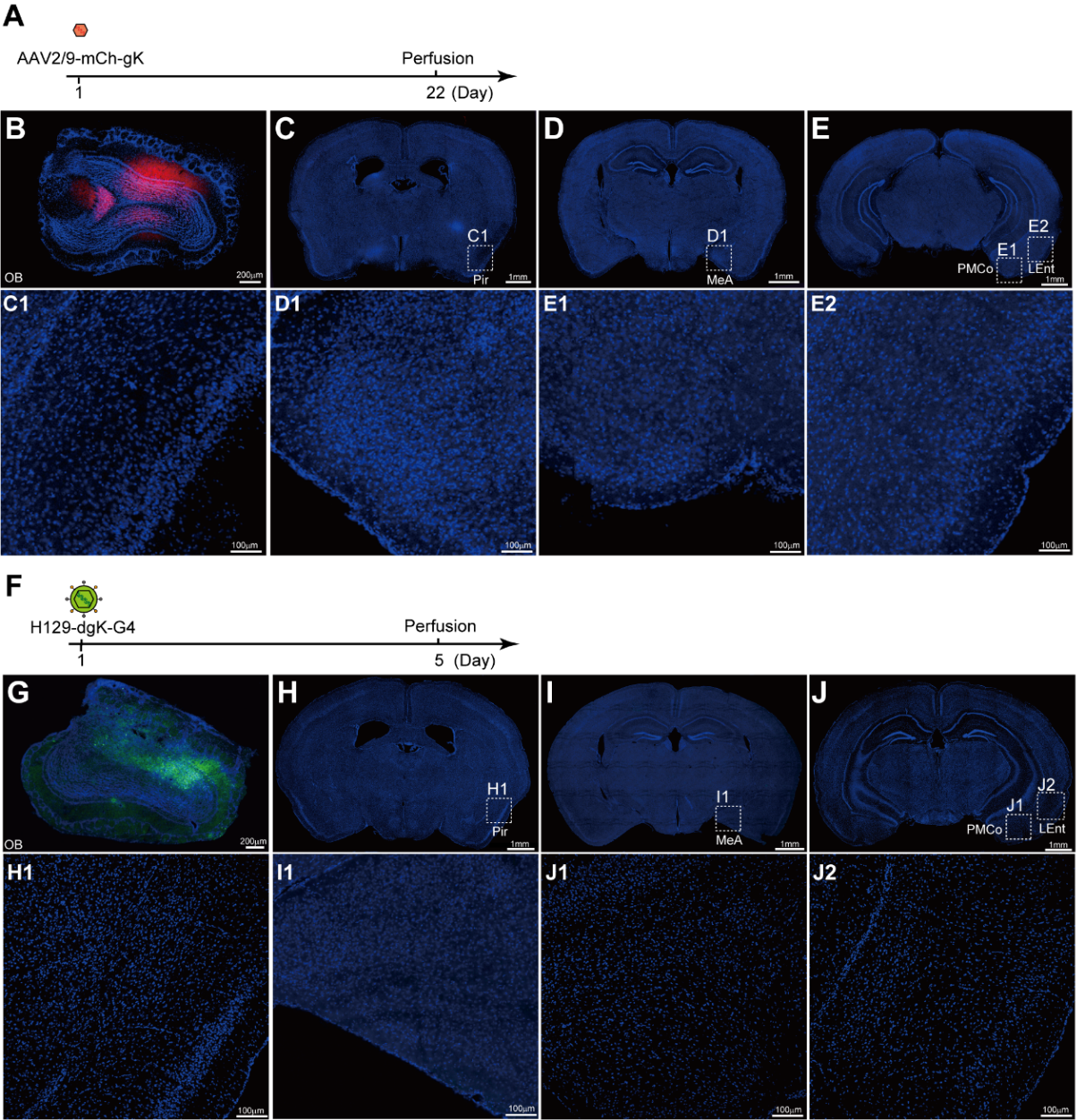
**

**Figure S4.** Control of OB circuit tracing

AAV2/9-mCh-gK (A) or H129-dgK-G4 (F) was injected into the OB alone, and the brains were collected for observation at the indicated times. Representative images of the injection site (B and G) and the downstream regions (C-E and H-J) are shown. The boxed regions are displayed correspondingly with higher magnification.

**Figure S5**

**
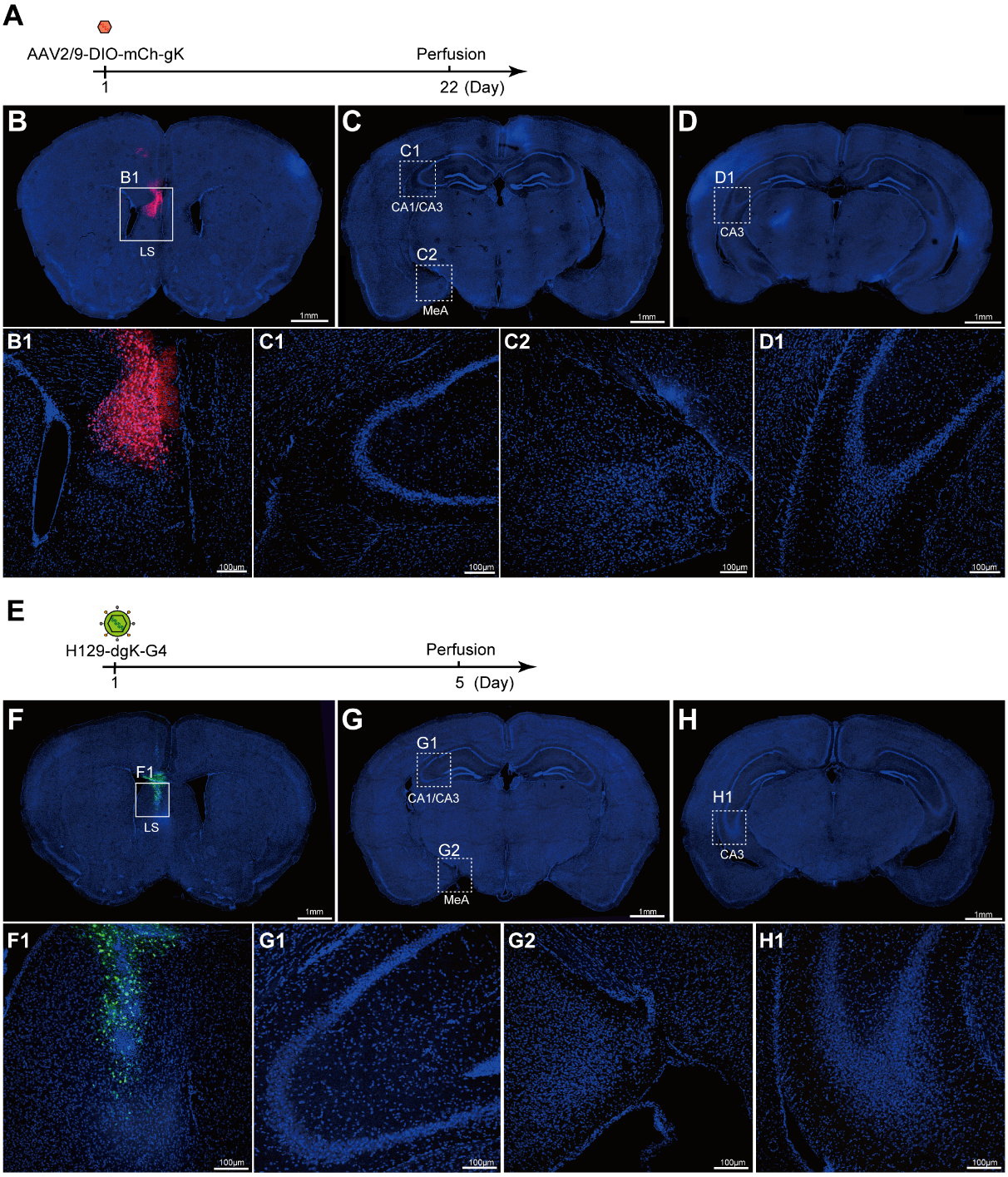
**

**Figure S5.** Control of starter specific LS circuit tracing

AAV2/9-mCh-gK (A) or H129-dgK-G4 (E) was injected into the LS alone, and the brains were collected for observation at the indicated times. Representative images of the injection site (B and E) and the downstream regions (C-D and G-H) are shown. The boxed regions are displayed correspondingly with higher magnification.

**Figure S6**

**
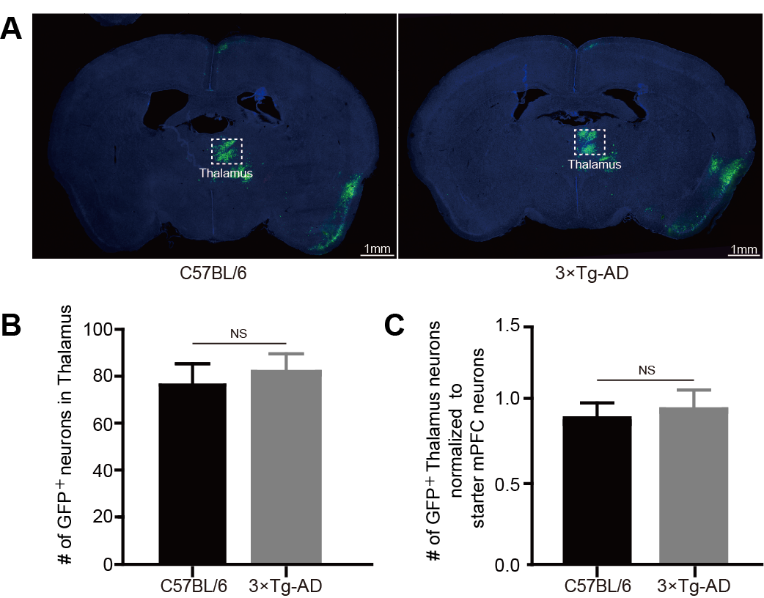
**

**Figure S6.** Quantitative comparison of mPFC-thalamus connections between Alzheimer's disease and control mice by tracing with H129-dgK-G4.

Thalamic nuclei of the same wildtype C57BL/6 and 3×Tg-AD mice shown in Fig.7 were examined, and the representative images of the GFP-labeled neurons in the thalamus (indicated by the dashed box) are shown (A). The GFP-labeled postsynaptic neurons in the thalamus were counted in three mice for each group (B), and the statistical significance was analyzed by LME. The average ratio of GFP^+^ thalamic neurons to mPFC starter neurons of each mouse was calculated (C), and the statistical significance was analyzed by Student’s t-test. NS, not significant.
